# MicroRNA profiles in hippocampal granule cells and plasma of rats with pilocarpine-induced epilepsy – comparison with human epileptic samples

**DOI:** 10.1038/srep14143

**Published:** 2015-09-18

**Authors:** Paolo Roncon, Marie Soukupovà, Anna Binaschi, Chiara Falcicchia, Silvia Zucchini, Manuela Ferracin, Sarah R. Langley, Enrico Petretto, Michael R. Johnson, Gianluca Marucci, Roberto Michelucci, Guido Rubboli, Michele Simonato

**Affiliations:** 1 Department of Medical Sciences, Section of Pharmacology and Neuroscience Center, University of Ferrara, Italy; 2 National Institute of Neuroscience, Italy; 3 Laboratory for Technologies of Advanced Therapies (LTTA), University of Ferrara, Italy; 4 Department of Morphology, Surgery and Experimental Medicine, Section of Pathology, Oncology and Experimental Biology, University of Ferrara, Italy; 5 Division of Brain Sciences, Imperial College London, Charing Cross Hospital,UK; 6 Medical Research Council (MRC) Clinical Sciences Centre, Imperial College London, Hammersmith Hospital, UK; 7 Department of Biomedical and NeuroMotor Sciences (DiBiNeM), Section of Pathology, Bellaria Hospital, Bologna, Italy; 8 IRCCS Institute of Neurological Sciences, Section of Neurology, Bellaria Hospital, Bologna, Italy; 9 Danish Epilepsy Center, Filadelfia/University of Copenhagen, Dianalund, Denmark

## Abstract

The identification of biomarkers of the transformation of normal to epileptic tissue would help to stratify patients at risk of epilepsy following brain injury, and inform new treatment strategies. MicroRNAs (miRNAs) are an attractive option in this direction. In this study, miRNA microarrays were performed on laser-microdissected hippocampal granule cell layer (GCL) and on plasma, at different time points in the development of pilocarpine-induced epilepsy in the rat: latency, first spontaneous seizure and chronic epileptic phase. Sixty-three miRNAs were differentially expressed in the GCL when considering all time points. Three main clusters were identified that separated the control and chronic phase groups from the latency group and from the first spontaneous seizure group. MiRNAs from rats in the chronic phase were compared to those obtained from the laser-microdissected GCL of epileptic patients, identifying several miRNAs (miR-21-5p, miR-23a-5p, miR-146a-5p and miR-181c-5p) that were up-regulated in both human and rat epileptic tissue. Analysis of plasma samples revealed different levels between control and pilocarpine-treated animals for 27 miRNAs. Two main clusters were identified that segregated controls from all other groups. Those miRNAs that are altered in plasma before the first spontaneous seizure, like miR-9a-3p, may be proposed as putative biomarkers of epileptogenesis.

Temporal lobe epilepsy (TLE) is the most common form of epilepsy in adults. It often develops secondary to an initial brain insult (e.g. trauma, tumor or stroke), after a latency period in which patients are apparently well. Thus, at-risk patients can often be identified, but it is currently impossible to predict who will actually develop epilepsy and who will not. Moreover, available antiepileptic drugs are symptomatic agents that are not useful in preventing the development of the disease and are ineffective in approximately one-third of patients with epilepsy^1^. Thus, there is an urgent need not only to develop new therapies for drug-resistant epilepsy, but also to identify antiepileptogenic therapies that can prevent the disease in at-risk individuals^1^,^2^,^3^. To do so, it is essential to better understand the mechanisms of epileptogenesis and drug-resistance. Moreover, the development of biomarkers for epileptogenesis following brain injury would facilitate clinical trials of novel antiepileptogenic therapies by allowing those patients at the greatest risk of epilepsy to be identified^1^,^4^,^5^. MicroRNAs (miRNAs) provide an opportunity as both therapeutic targets and biomarkers of epileptogenesis.

MiRNAs are small (~22 nt) non-coding RNAs that regulate the expression of target mRNAs at the post-transcriptional level^6^. Experimental evidence has demonstrated that miRNAs are involved in heterogeneous brain functions^7^,^8^ including neuroinflammation^9^, synaptic remodeling^10^, and neuronal death^11^. Thus, it can be hypothesized that specific miRNAs may regulate genetic programs leading to hyperexcitability in the brain and, as such, represent new therapeutic targets. Moreover, miRNAs are found in the blood, where their levels are affected by disease states^12^,^13^,^14^,^15^ and are therefore attractive candidates as biomarkers for stratifying patients at highest risk of epilepsy following brain injury^16^,^17^.

Recently, alterations in miRNA expression levels have been described both in the brain of epilepsy patients and in animal models of epilepsy. In humans, Kan and colleagues performed a genome-wide profiling of TLE, identifying a miRNA signature of hippocampal sclerosis^18^. Likewise, a recent study demonstrated the implication of miR-487a in granule cell pathology^19^. In animal models, changes in miRNA expression levels in the hippocampus have been reported in multiple studies in the latency period after an epileptogenic insult and in the chronic epileptic state^20^,^21^,^22^,^23^,^24^. Unfortunately, all these studies have multiple limitations. First, the brain tissue was the whole hippocampus or a mechanically-dissected hippocampal sub-region in most studies, which implicates not only great heterogeneity in terms of cell composition, but also a different representation of different cell types in controls and epileptic samples, as the epileptic hippocampus is characterized by cell loss and astrocytosis. Only Zucchini *et al.*^19^ microdissected a specific cell population (the granule cells), but the comparison was between epileptic samples with or without granule cell pathology, i.e. no healthy controls. Second, human studies lacked a proper control, in that the epileptic tissue was from surgeries and the control from autopsies. Third, no detailed evaluation of the changes in the course of disease was performed. Forth, blood samples were not collected and analyzed, except for Gorter *et al.*^24^

The aim of this study was to fill the gaps left by the previous ones of this kind. Specifically, we performed a systematic evaluation of the miRNAome in a specific cell population of the hippocampus (the laser microdissected granule cell) and in plasma samples, at multiple time-points in the course of pilocarpine-induced epilepsy in rats: early and late latency, at the time of the first spontaneous seizure and in the chronic period. Further, we compared results from rats in the chronic phase of epilepsy with post-mortem human epileptic and control granule cell samples.

## Results

### Granule cells

#### miRNA clustering

**Figure 1:**
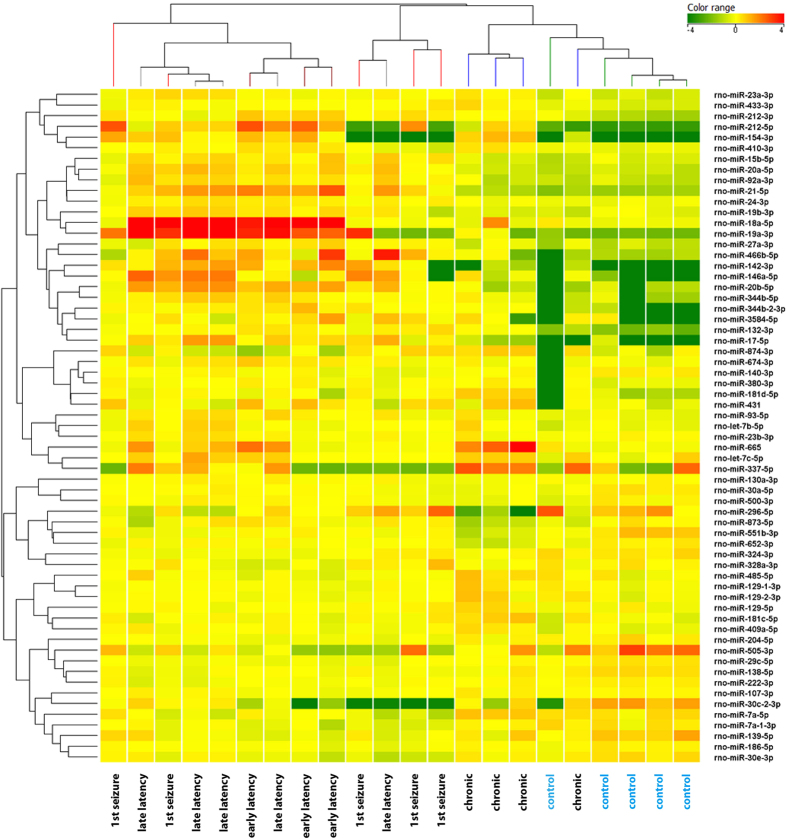
Cluster analysis of miRNAs differentially expressed in the granule cell layer (GCL) at different time points in the course of pilocarpine-induced epilepsy. Heat map representation of the average expression of the 63 differentially expressed miRNAs in the GCL of epileptic and control animals. Each column represents an individual animal and each row represents one miRNA, as indicated. Colors represent the expression level fold change: higher-red, lower-green. Analysis was performed on differentially expressed miRNAs (FDR > 10%), using the Person correlation; for cluster image preparation an additional step of normalization on gene median across all samples was added.

We first evaluated miRNA expression in the laser-microdissected granule cell layer (GCL) of rats sacrificed at multiple time-point following pilocarpine-induced SE: 4 and 7 days after SE, 12 h after the first spontaneous seizure and 50 days after the first spontaneous seizure. To detect changes in miRNA expression levels regardless of the time point, we first analyzed differential miRNA expression levels in the GCL between control and epileptic rats. We detected significant changes in the expression of 63 miRNAs (Supplementary Table S1). Hierarchical clustering of all samples performed with these 63 miRNAs revealed three main clusters that separated the control and chronic phase groups from rats sacrificed during latency (4 and 8 days after SE) and from those sacrificed after the first spontaneous seizure (Fig. 1). However, control and chronic formed distinguishable sub-clusters.

Changes in expression were also separately analyzed for each time point. As compared with controls, we detected significant changes in 33 miRNAs during latency (4 and 8 days after SE), 33 miRNAs at the time of the first spontaneous seizure, and 25 miRNAs in the chronic period (Supplementary Tables S2–S4).

#### miRNA expression pattern

**Figure 2:**
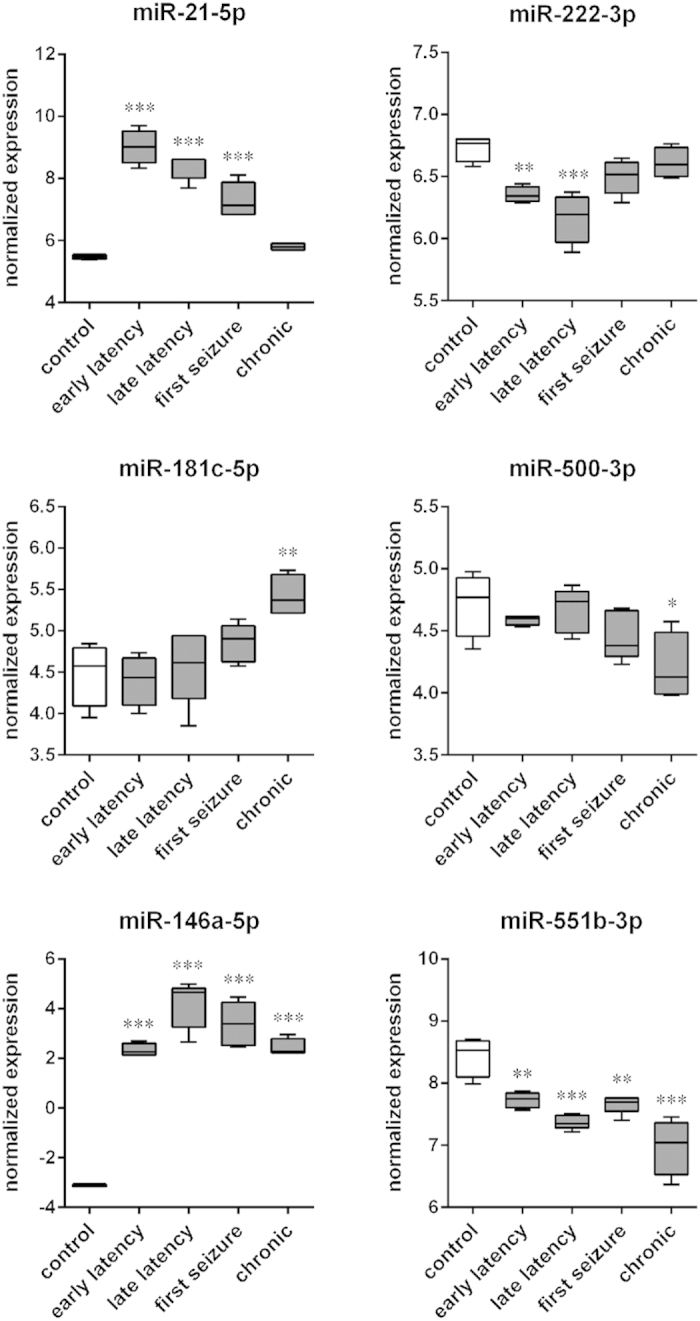
Time course patterns of miRNA expression in the rat granule cell layer (GCL). The boxplots depict the time course of expression for 6 of the miRNAs identified as significant with a false discovery rate (FDR) < 10% in the GCL by using one-way ANOVA. Each boxplot represents 4 animals. These miRNAs were chosen as representative of the different patterns that were observed: up-regulation (miR-21-5p) or down-regulation (miR-222-3p) during latency; up-regulation (miR-181c-5p) or down-regulation (miR-500-3p) in the chronic period; up-regulation (miR-146a-5p) or down-regulation (miR-551b-3p) in the entire course of the disease. The time courses of the other significantly modified miRNAs are shown in Supplementary Figs. S2, S3, S4, S5 and S6. *p < 0.05; **p < 0.01; ***p < 0.001; Tukey’s test.

Variations in miRNAs levels in the GCL at different time points were then analyzed in detail. Group to group comparison revealed 6 different miRNA expression patterns in the GCL. First, a subgroup of miRNAs (miR-15b-5p, miR-17-5p, miR-18a-5p, miR-19a-3p, miR19b-3p, miR-20a-5p, miR-20b-5p, miR-21-5p, miR-23b-5p, miR-24-3p, miR-27a-3p, miR-92a-3p, miR-93-5p, miR-142-3p, miR-344b-2-3p, miR-431, miR-466b-5p and miR-674-3p) displayed increased expression levels during latency (4 and 8 days after SE), decreased their expression levels at the time of the first spontaneous seizure and returned to control levels in the chronic phase (Fig. 2, Supplementary Fig. S1). All these miRNAs displayed a peak in expression levels 8 days after SE, except for miR-21-5p, miR-27a-3p, miR-142-3p and miR-674-3p that peaked earlier (4 days after SE). Another subgroup of miRNAs displayed an opposite pattern, i.e. decreased expression during latency: miR-7a-1-3p, miR-107-3p, miR-138-5p, miR-139-3p, miR-186-5p, miR-204-5p, miR-222-3p, miR-324-3p and miR-505-3p were significantly decreased during latency (peak at 4 days after SE), then gradually returned to control levels (Fig. 2, Supplementary Fig. S2).

Some miRNAs (miR-129-1-3p; miR-129-2-3p, miR-129-5p, miR181c-5p, miR181d-5p, miR-409a-5p, miR-655 and miR-874-3p) were up-regulated (Fig. 2, Supplementary Fig. S3A), whereas others (miR-296-5p, miR-500-3p and miR-652-3p) were down-regulated only in the chronic phase, while not being significantly altered during latency (Fig. 2, Supplementary Fig. S3B). Finally, other subsets of miRNAs were either up-regulated (miR-23a-3p, miR132-3p, miR-146a-5p, miR-154-3p, miR-181d-5p, miR-212-3p, miR-212-5p, miR-344b-5p, miR-380-3p, miR-410-3p, miR-433-3p and miR-3584; Fig. 2, Supplementary Fig. S4), or down-regulated (miR-29c-5p, miR-30a-5p, miR-30c-2-3p, miR-30e-3p, miR-138-5p, miR-140-3p, miR-551b-3p and miR-652-3p; Fig. 2, Supplementary Fig. S5) during all phases of the disease.

#### Network analysis

**Figure 3:**
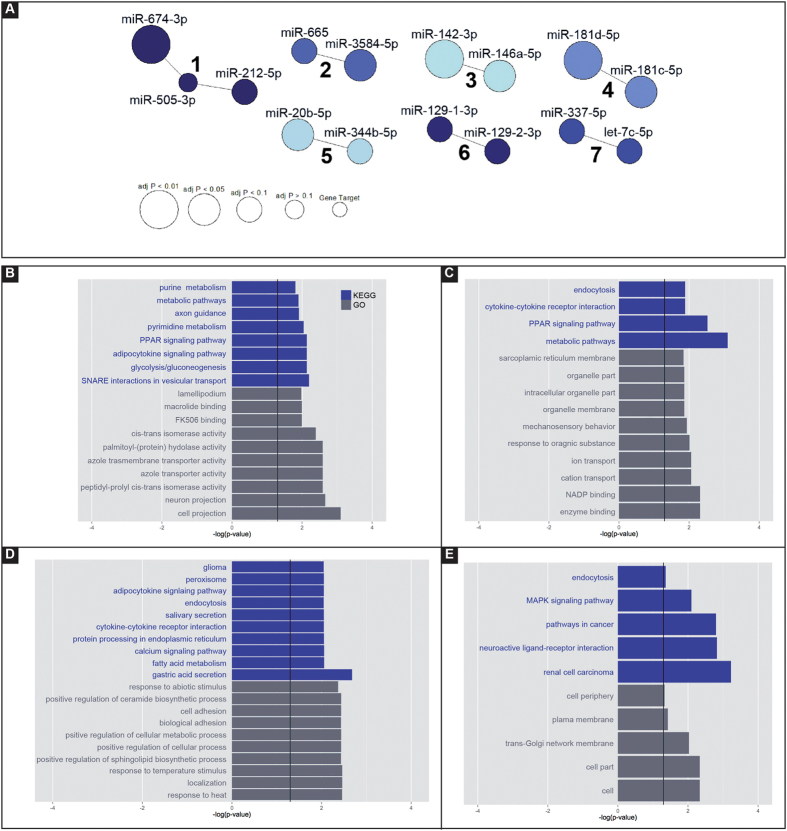
Network analysis. Predicted gene targets for each miRNA dys-regulated during latency (4 and 8 days after SE), obtained using the mirDB and TargetScan algorithms, were filtered with all miRNAs expressed in the dentate gyrus of rats (database GSE49850). (**A**) Clusters of miRNAs identified at 10% false discovery rate (FDR) level. (**B**) Cluster 1. (**C**) Cluster 3; (**D**) Cluster 4. (**E**) Cluster 5. Gene ontology (GO) and Kyoto Encyclopedia of Genes and Genomes (KEGG) analysis were performed using webgestalt, p < 0.05.

We then performed a network analysis of miRNAs whose expression was significantly altered during latency, because pathways identified in this manner may play a role in epileptogenesis. Targets for each miRNA were obtained using the mirDB and TargetScan algorithms and filtered to include those which were expressed in the rat dentate gyrus. Seven significant clusters of miRNAs were identified at 10% false discovery rate (FDR) level (Fig. 3A). GO/KEGG enrichment highlighted five of them as having a potential role in epileptogenesis.

Cluster 1, composed by miR-674-3p, miR-505-3p and miR-212-5p, was up-regulated during epileptogenesis (4 and 8 days after SE). Target analysis identified eight significant pathways: purine metabolism (adjusted P = 0.016); metabolic pathways (adjusted P = 0.013); axon guidance (adjusted P = 0.013); pyrimidine metabolism (adjusted P = 0.0091); peroxisome proliferator-activated receptors (PPAR) signaling pathway (adjusted P = 0.0074); adipocytokine signaling pathway (adjusted P = 0.0074); glycolysis and gluconeogenesis (adjusted P = 0.0074); SNAp REceptor (SNARE) interactions in vesicular transport (adjusted P = 0.0064; Fig. 3B). “Axon guidance” and “SNARE interactions in vesicular transport” were the categories most likely to play a role in latency. Two target genes per pathway were highlighted: the synaptosomal-associated protein 29 kDa (Snap 29) and the vesicle-associated membrane protein 3 (Vamp 3) in the axon guidance pathway; the ephrin B1 (Efnb1) and the chemokine ligand 12 (Cxcl12) in the SNARE interactions in vesicular transport pathway (Supplementary Fig. S6A).

Cluster 3 was composed by two up-regulated microRNAs, miR-142-3p and miR-146a-5p. This cluster was associated with four different pathways including endocytosis (adjusted P = 0.013), cytokine-cytokine receptor interaction (adjusted P = 0.013), PPAR signaling pathway (adjusted P = 0.003) and general metabolic pathway (adjusted P = 0.0008; Fig. 3C). Two target genes in cytokine-cytokine receptor interaction pathway were highlighted; the transforming growth factor β receptor 1 (Tgfbr1) and the growth hormone receptor (Ghr; Supplementary Fig. S6B).

Cluster 4 included miR-181c-5p and miR-181d-5p. The KEGG analysis identified multiple pathways associated with the target genes of these two miRNAs. In particular, the neurotrophin signaling pathway (adjusted P = 0.016), with tumor necrosis factor receptor superfamily, member 11b (Tnfrsf11b), protein kinase C, delta (Prkcd) and calmodulin 1 (Calm1); and the cytokine-cytokine receptor interaction pathway (adjusted P = 0.009), with the platelet-derived growth factor receptor, alpha polypeptide (Pdgfra) and the chemokine ligand 10 (Cxcl10; Fig. 3D and Supplementary Fig. S6C).

MiR-344b-5p and miR-20b-5p were in cluster 5. They were associated with different cancer related pathways, endocytosis (adjusted P = 0.044), MAPK signaling (adjusted P = 0.0081) and neuroactive ligand-receptor interaction (adjusted P = 0.0015; Fig. 3E). Target genes involved in neuroactive ligand-receptor interaction category were the glycine receptor alpha 2 (Glra2), the gamma-aminobutyric acid B receptor 2 (Gabbra2), the galanin receptor 1 (GalR1) and the glutamate receptor ionotropic AMPA 2 (Gria2; Supplementary Fig. S6D).

Interestingly, this combinatorial analysis revealed that some significant clusters of miRNAs might correlate with identical pathways.

#### RT-qPCR

**Figure 4:**
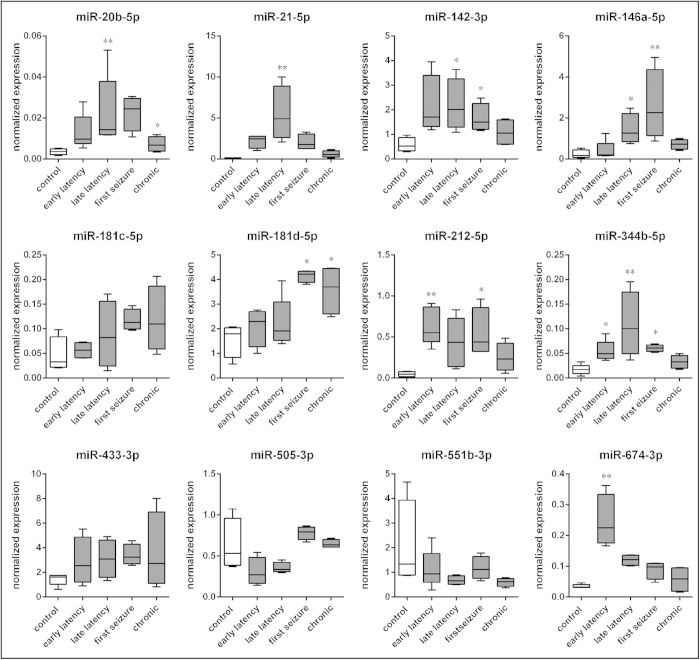
Relative expression of selected miRNAs in the granule cell layer, as detected using qPCR. Five rats per group. *p < 0.05; **p < 0.01; Tukey’s test.

A large subset of miRNAs whose expression was significantly modulated in the GCL in our model and that was identified in significant clusters through network analysis was validated using RT-qPCR in a different cohort of cases. Overall, the data were highly consistent with those obtained using microarray. In fact, the expression patterns of miR-20b-5p, miR-142-3p, miR-181d-5p, miR-212-5p, miR-344b-5p and miR-674-3p were identical to those observed using the microarray, and those of miR-21-5p and miR-146a-5p were very similar, although not identical (Fig. 4). Even if displaying the same patterns observed with the microarray, the expression levels of mir-181c-5p, miR-433-3p, miR-505-3p and miR-551b-3p were not significantly different from controls (Fig. 4).

#### Human tissue

Next, we asked what the relevance of these findings could be for human epilepsy. In humans, comparison can only be made between chronically epileptic and non-epileptic controls. However, a technical hurdle exists with human studies, because the only available human controls are from autopsies, whereas the epileptic tissue is generally from surgical resections. Thus, we first wanted to ensure that the comparison of autoptic and bioptic material was valid. We performed miRNA microarray on laser-microdissected GCLs from 10 epileptic patients who underwent surgery, two autopsies of epileptic patients and 10 aged-matched autopsy controls (epileptic: age 70 and 46, both females; non-epileptic: age 47 ± 7, range 34–57, 3 males, 7 females; all 12 dead by acute pulmonary or cardiac disease). Unfortunately, the two autoptic epileptic did not segregate with the other epileptic as expected, but with the control autoptic. This indicates that the tissue origin (i.e. bioptic or autoptic) had greater importance than the disease background (data not shown, manuscript in preparation). Therefore, here we restricted the comparison between the 2 epileptic and the 10 non-epileptic autoptic samples.

We compared the miRNA species modulated in chronic epileptic rats and in human cases. We identified four miRNAs (miR-21-5p, miR-23a-5p, miR-146a-5p and miR-181c-5p) that were up-regulated in both epileptic humans and rats (Table 1). Moreover, the comparison displayed similar changes in expression levels between epileptic (human cases and rats) and controls, except for miR-146a-5p, that increased more in rats than in humans (fold change equals 21.24 in rats and 3.63 in humans).

### Plasma

Finally, to investigate the possibility of using miRNAs in peripheral blood as potential biomarkers of epileptogenesis, we analyzed plasma samples collected from the experimental and control rats for the study of the hippocampal GCL.

**Figure 5:**
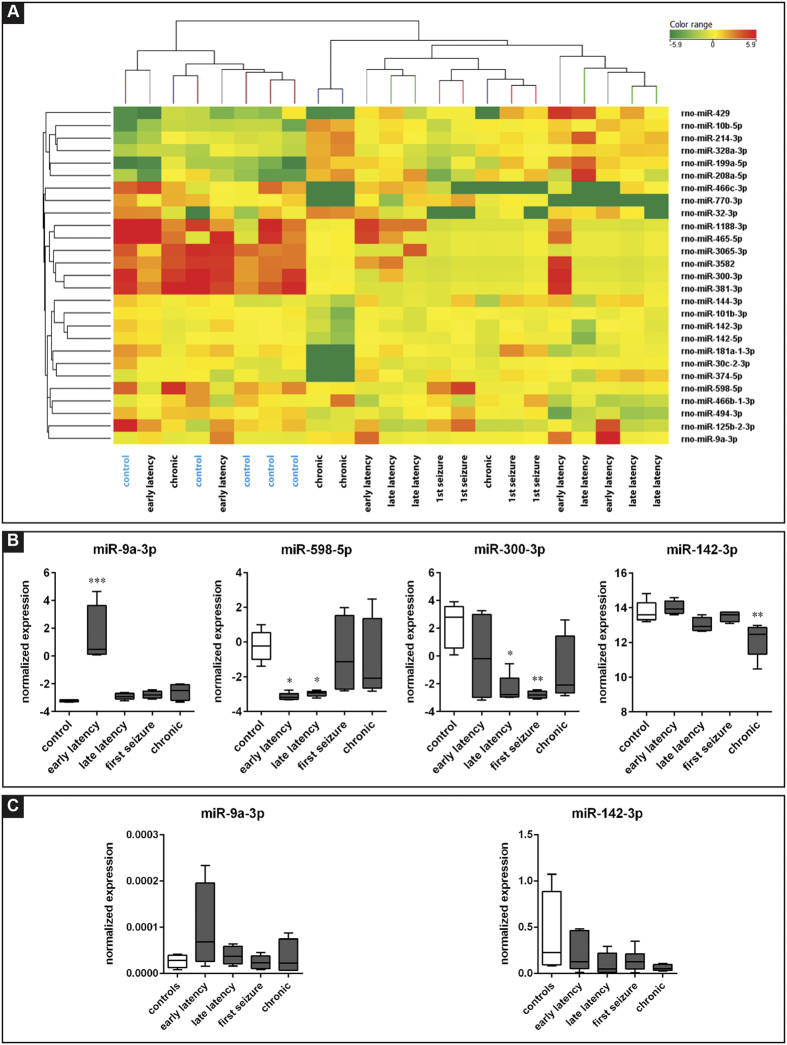
Plasma miRNA levels. (**A**) Heat map representation of the average levels of the 27 differentially expressed miRNAs in the plasma of epileptic and control animals. Each column represents an individual animal and each row represents one miRNA, as indicated. Colors represent the expression level fold change: higher-red, lower-green. Analysis was performed on differentially represented miRNAs using the Person correlation. (**B**) The boxplots depict the time course for 4 of the miRNAs identified as significant by using one-way ANOVA. Each boxplot represents 5 animals. These miRNAs were chosen as representative of the different patterns that were observed: up-regulation (miR-9a-5p) or down-regulation (miR-598-5p) during latency, down-regulation in the late latency - first spontaneous seizure period (miR-381-3p) and down regulation in the chronic stage (miR-142-5p). (**C**) Relative levels of miR-9a-5p and miR-142-3p, as detected using qPCR. Five rats per group. *p < 0.05; **p < 0.01; ***p < 0.001; Tukey’s test.

This analysis revealed different levels between control and pilocarpine-treated animals in one miRNAs: only miR-91-3p showed significant differential expression at a 10% FDR level (adjusted P = 0.08). This may be due to the larger variability found in circulating miRNAs levels as compared to other tissues^25^. Therefore, to gain an exploratory view of the miRNA expression patterns in plasma, we relaxed the significance threshold to an unadjusted P < 0.05, which resulted in 27 miRNAs of interest (Supplementary Table S5). Hierarchical clustering also generated two main clusters that separated controls from all other groups, with the exception of two animals in early latency group (4 days after SE) and one in the chronic phase group, which segregated with the controls (Fig. 5A). Within the cluster including all pilocarpine animals, rats belonging to the single experimental groups displayed a clear tendency to form distinct sub-clusters.

Within these 27 miRNAs, we detected 4 different expression patterns in the plasma of rats sacrificed at different time points of the disease history. MiR-9a-3p displayed highly increased levels at the earlier stage of latency (4 days after SE), then normalized to control levels at the following time points (Fig. 5B; Supplementary Fig. S7A). A second pattern was about the opposite: miR-466b-1-3p, miR-494-3p and miR-598-5p displayed significantly decreased plasma levels during latency, and a tendency to return to the control levels in the chronic stage (Supplementary Fig. S7B). Another couple of miRNAs (miR-32-3p and miR-300-3p) was down-regulated around the time of the first spontaneous seizure (Supplementary Fig. S7C). Finally, miR-30c-2-3p, miR-101b-3p, miR-142-3p, miR-142-5p, miR-181a-1-3p, miR-374-5p, miR-466c-3p, miR-1188-3p, miR-3065-3p and miR-3582 were significantly down-regulated in the chronic stage (Supplementary Fig. S7D).

The only miRNA dysregulated both in the GCL and in plasma was miR-142-3p. The patterns of dysregulation, however, were totally different: miR-142-3p was up-regulated in the GCL during latency and down-regulated in plasma in the chronic period. MiR-9a-3p and miR-142-3p were chosen for RT-qPCR validation in the same cohort of samples, displaying the same patterns observed in the microarray, even if they did not reach statistical significance.

## Discussion

The main findings of the present study are the following. First, the overall analysis of the rat GCL revealed three main clusters of miRNAs that separated the control and chronic phase groups from rats sacrificed during latency (4 and 8 days after SE) and from those sacrificed after the first spontaneous seizure. This identifies a distinct miRNA expression pattern associated with the latency period between brain injury and first spontaneous seizure that may reveal epileptogenic pathways. Second, the chronic phase was accompanied by significant alterations in miRNA expression in the rat GCL, and comparison with data from epileptic patients identified several miRNAs (notably miR-21-5p, miR-23a-5p, miR-146a-5p and miR-181c-5p) that were up-regulated in both human and rat epileptic hippocampus. MiRNAs whose expression is altered in the GCL may be implicated in the mechanisms of epileptogenesis and/or in the generation of seizures, and may therefore represent new therapeutic targets. Third, analysis of plasma samples revealed that, while at relaxed threshold, 27 miRNAs were able to discriminate the controls from all other groups. Those miRNAs that are altered in plasma before the first spontaneous seizure, like miR-9a-3p, may be worth further investigation into their use as potential biomarkers of epileptogenesis.

The miRNA expression analysis revealed interesting overlaps between our work here and the two previously published studies focusing on the dentate gyrus (DG)^23^,^24^, both in terms of miRNAs modulated in the latency and in the chronic phase, even if: 1) different epilepsy models were employed in these other studies, namely amygdala and perforant path stimulation-induced SE; 2) the DG was mechanically dissected from other hippocampal regions in these other studies, and therefore the results are the combination of the varying alterations in miRNA levels occurring in multiple cell populations (in contrast with our laser-dissection approach, that ensures a nearly pure granule cell preparation). Other studies^20^,^21^,^22^ used the whole hippocampus, emphasizing the latter limitation, and therefore will not be used for comparison in the frame of the present discussion.

Commonalities with other studies are particularly interesting, because they may represent disease-specific, rather than model-specific alterations. Since the pathophysiological implications would be very different, we will here discuss separately findings related to latency (early and late latency in the present study) and findings related to the chronic state that follows diagnosis in humans (first seizure and chronic murine groups plus the human cases in this study). Finally, we will discuss the data obtained from peripheral blood.

### Latency

Our study and other relevant data sets^23^,^24^ identified the up regulation of three miRNAs (miR-21-5p, miR-212-3p and miR-132-3p) during latency. Furthermore, we and Gorter *et al.*^24^ observed the up-regulation of miR-17-5p, miR-20a-5p, miR-23a-3p and the down-regulation of miR-139-5p, whereas we and Bot *et al.*^23^ observed the down-regulation of miR-551b-3p.

Alterations on these miRNA levels suggest a role in the mechanism of epileptogenesis, a concept that is supported by analysis of their potential targets. For example, the up-regulation of miR-21-5p may be involved in the initiation of the cell signaling pathway associated with epilepsy^26^. Moreover, mutations in the myocyte enhancer factor 2C (MEF2C) gene, a neuronal transcription factor that may have a role in neuronal dysfunction and neurodegeneration^27^, has been observed in patients with epilepsy^28^ and has been identified as a target of miR-21-5p and miR-21-3p^27^. Sestrin 1 (SESN1), a predicted target of miR-21-5p, is down-regulated during latency after amygdala stimulation^23^. This finding, combined with the evidence that another member of the sestrins family, SESN3, controls a proconvulsant transcriptional program in human TLE^29^, suggests a correlation between miR-21-5p and the mechanisms that lead to seizures onset.

Further evidence for a direct involvement of the identified miRNAs in the mechanisms of epileptogenesis derived from network analysis. Our network analysis, based on miRNAs dysregulated during latency in the GCL, highlighted the presence of 4 clusters of miRNAs. Cluster 3 (that includes miR-142-3p and miR-146a-5p) and cluster 4 (including miR-181c-5p and miR-181c-5p) are connected to the “cytokine-cytokine receptor interaction” signaling pathway, which suggests a neuroinflammatory role for those miRNAs. In keeping with this idea, evidence in the literature supports that miR-146a-5p regulates the astrocyte-mediated inflammatory response by influencing IL-1β, IL-6 and COX-2 signaling^24^,^30^. Cluster 4, together with cluster 1 (that includes miR-674-3p, miR-505-3p and miR-212-5p) and cluster 5 (including miR-144b-5p and miR-20b-5p), may also strongly influence neuronal activity. In fact, their predicted targets in the rat DG are involved in axon guidance and SNARE interaction (cluster 1), neurotrophin signaling (cluster 4) and neuroactive ligand receptor (cluster 5). Axon guidance has been linked to epilepsy, the sprouting of mossy fibers (the axons of granule cells) being the best characterized of axonal reorganization in TLE. Although epilepsy-associated mossy fiber sprouting has been extensively studied^31^,^32^,^33^, its underlying molecular mechanisms are still poorly understood, and the present findings suggest a role for this cluster of miRNAs. Interestingly, the predicted targets of cluster 5 include the gamma-aminobutyric acid B receptor 2 (Gabbra2), the galanin receptor 1 (GalR1) and the glutamate receptor ionotropic AMPA 2 (Gria2), all genes whose products are implicated in the regulation of excitability and in epilepsy. Specifically, 1) a polymorphism of Gabbra2 is associated with mTLE^34^; 2) GalR1 deletion exacerbates hippocampal neuronal loss after kainate administration in mice^35^, and GalR1 knockout mice exhibit spontaneous epilepsy, abnormal EEGs, and altered inhibition in the hippocampus^36^; 3) Gria2 in the hippocampus is related to the mechanisms of seizure and neurodegeneration^37^. Thus, this cluster of miRNAs may play a role as a master regulator of multiple mechanisms of epileptogenesis. Functional studies aimed at establishing the functional role of these miRNAs in TLE will represent the obvious continuation of this study.

Most of the miRNAs up-regulated during latency in the GCL displayed pick expression levels in the late phase of latency (7 days after SE), but a subset (miR-21-5p, miR-674-3p and miR-3564-5p) was increased the most 4 days after SE. It can be hypothesized that these changes correspond to different “waves” of the epileptogenesis process, which may represent different windows of opportunity for therapy^38^.

### Chronic period

Regarding the chronic group, both the present dataset and the other two that we considered relevant to this Discussion^23^,^24^ identified the up-regulation of miR-21-5p, miR-23a-5p and miR-146a-5p. Up-repulation of these miRNAs has been reported also in other studies^21^,^39^,^40^. Moreover, we and Gorter *et al.*^24^ observed the up-regulation of miR-212-5p, whereas we and Bot *et al.*^23^ observed the up-regulation of miR-433-3p.

MiR-23a-5p has been proven to be a regulator of cell growth and apoptosis^41^. Furthermore, it has been identified as a negative regulator of lamin B1, thereby contributing to the process of oligodendroglia development and myelin formation^42^. MiR-146a-5p is strictly associated with the astrocyte-associated immune response both in epileptic patients and *in vivo* models^24^,^39^.

As described, we identified different expression patterns of miRNAs that were up or down-regulated at the time of the first seizure and during the chronic period, showing slight but significant differences between the two phases. Continuing modifications in the expression pattern of miRNAs in the course of chronic epilepsy support the hypothesis that epileptogenesis is a dynamic process that continues even after the initial diagnosis of the disease, i.e. after the initial spontaneous seizures^1^.

The comparison between chronic epileptic rats and the human cases identified four miRNAs (miR-21-5p, miR-23a-5p, miR-146a-5p and miR-181c-5p) that are similarly up-regulated in expression levels in both species. Interestingly, miR-23a-5p and miR-146a-5p are in common with the other two rat data sets that were taken as primary comparator in this Discussion^23^,^24^. As for miR-181c-5p, it was highlighted by network analysis in cluster 4, and implicated in cytokine-cytokine receptor interaction and in the inflammatory response. Thus, even if preliminary being based on a small number of patients, human data appear to confirm those obtained in animal models, suggesting that animal data may be representative of the human situation also under conditions (latency) that cannot be directly explored in humans.

### Blood

There is great need of diagnosis tools to identify those individuals, among those at risk, that will actually develop epilepsy or to stratify epileptic patients with different prognosis. Changes in extracellular miRNA levels have been observed in association with neurological diseases in various body fluids including plasma, putting forward the concept that miRNAs may represent noninvasive or minimally invasive, clinically useful biomarkers^16^,^17^,^24^, even if the biological function of plasmatic miRNAs is still obscure (it has been hypothesized that they represent a form of cellular communication whereby cells can interact at distance by transfer of exosomes or microvesicles containing miRNAs through the systemic circulation^43^,^44^).

Two previous studies performed microarrays on blood samples. One described changes in circulating miRNA levels 24 h after ischemic stroke, intracerebral hemorrhage and kainate seizures^45^. The other^24^ performed the analysis in plasma samples obtained from the trunk blood, reporting an increase in miR-142-5p levels during the acute phase, miR-21-5p in the early stage and of miR-146a-5p in the chronic stage, that reflect parallel changes in miRNAs expression observed in the brain. Unfortunately, the complete dataset has not been made available by this study, and it is therefore unclear if all plasma miRNAs or only those 3 change in the same manner in plasma and in the brain.

Here, we studied the expression levels of circulating miRNAs in plasma separated by blood collected from animals through an intracardiac withdrawal, a technique that avoids contamination with others cell populations. Analysis of plasma samples revealed different levels between control and pilocarpine-treated animals for 27 miRNAs that segregated controls from all other groups. We identified a single miRNA, miR-142-3p that was altered both in plasma and in the brain, but the pattern of expression was different in that it was down-regulated in the former and up-regulated in the latter. Based on our data, therefore, it seems unlikely that changes in circulating miRNAs levels are correlated to the changes in miRNAs expression in the brain.

Although it is impossible, at the current stage of knowledge, to speculate on the biological significance of changes in plasma, it can nonetheless be proposed that those miRNAs that are altered in plasma before the first spontaneous seizure, like miR-9a-3p, are putative biomarkers of epileptogenesis. Further studies are needed to gain insight into the origin and the biological significance of circulating miRNAs, and to understand their functional role in TLE and other neurological disorders. The present data, however, are relevant to the identification of biomarkers that can predict the development of epilepsy in at-risk subjects.

In conclusion, the present results identify a distinct miRNA expression pattern associated with latency in the rat pilocarpine model and reveal putative epileptogenic pathways, suggesting that miRNAs represent attractive therapeutic targets for the prevention of epilepsy. Moreover, we found overlap human and rat databases in the chronic stage, supporting the notion that animal data are representative of the human situation. There are many commonalities between our data and other publicly available datasets obtained from *in vivo* models of TLE. Those miRNAs whose expression changes are in common through different datasets are particularly attractive candidates for further investigation, being disease-, rather than model-specific. Finally, results obtained from rat plasma samples suggest that circulating miRNAs may be used as biomarkers of epilepsy. The major hits identified in this study, like miR-9a-3p, should be now challenged in other epilepsy models in which only a subset of animals develop spontaneous seizures, in order to verify that they can actually stratify subjects that will or will not develop the disease.

## Methods

### Animals

Male Sprague-Dawley rats (250–350 g; Harlan, Italy) were used for pilocarpine experiments. They were housed under standard conditions: constant temperature (22–24 °C) and humidity (55–65%), 12 h light/dark cycle, free access to food and water. Procedures involving animals and their care were carried out in accordance with European Community, national and local approved guidelines, laws and policies. All experimental protocols were approved by the University of Ferrara Ethical Committee for Animal Experimentation and by the Italian Ministry of Health. All animals were (i) acclimatized to laboratory conditions for at least 1 h before the start of the experiment, (ii) used only once during the pilocarpine protocol and (iii) euthanized at different times points by anesthetic overdose. The number of animals was kept as small as possible and the ARRIVE (Animal Research: Reporting *In Vivo* Experiments^46^) have been followed.

### Surgery

Surgery was carried out to implant the electrodes for EEG recordings. Rats were secured to a stereotaxic apparatus, with the nose bar positioned at +5 mm, under ketamine/xylazine (43 and 7 mg/Kg i.p.) anesthesia. Anesthesia was then maintained using isoflurane (1.4% in air; 1.2 ml/min). A recording electrode was implanted into the right ventral hippocampus (A: −3.4 mm; L: +4.5 mm; P: +6.5 mm to bregma^47^). A reference electrode was placed on the skull. Rats were allowed 7 days to recover from surgery.

### Pilocarpine

Rats were randomly assigned to groups that received a single injection of methyl-scopolamine (1 mg/kg, s.c.) 30 min prior to pilocarpine (370 mg/kg, i.p.) or a single injection of methyl-scopolamine 30 min prior to vehicle (0.9% NaCl solution; control animals), and their behavior was observed by experienced researchers for at least 6 h thereafter. Within the first 20–25 min after pilocarpine injection, 83% of the animals developed seizures evolving into recurrent generalized convulsions (status epilepticus, SE). SE was interrupted 3 h after onset by administration of diazepam (20 mg/kg, i.p.). For 1–2 days following SE interruption, animals occasionally exhibited short-lasting seizures and lost weight (15–20%). Thereafter, they recovered and entered a period of apparent wellbeing (latency). However, one fourth of the animals that entered SE (i.e. 21% of those administered pilocarpine) died during SE or within 1–2 days.

Test and interspersed control animals were then randomly assigned to four experimental groups representing different phases of the natural history of the disease: early latency (4 days after SE), late latency (8 days after SE), first spontaneous seizure (11 ± 1 days after SE), chronic (50 days after the first spontaneous seizure). Inclusion criteria were (i) development of convulsive SE within 1 h after pilocarpine administration and (ii) weight gain within the first week after SE.

### Video and electroencephalography recordings

Video and video-EEG recordings were used to verify the hippocampal seizure onset and the disease progression in chronic animals. Convulsive seizures were assessed by 24/24-h video monitoring, performed using a digital video surveillance system DSS1000 (V4.7.0041FD, AverMedia Technologies, USA). Video monitoring was started approximately 6 h after pilocarpine administration (i.e. at the end of direct observation by the researchers – see above) and continued until day 5 when EEG recording was applied for proper identification of the first spontaneous seizure. Continuous video-EEG monitoring was started from day 5 after SE until the day of the first spontaneous seizure. Video-EEG monitoring (hardware system MP150 and software AcqKnowledge 4.3, all from Biopac, USA) was started at day 5 because, as previously reported^48^,^49^, we do not observe spontaneous seizures earlier than 8–9 days after pilocarpine administration under the experimental conditions employed in this study. EEG seizures were categorized and measured as periods of paroxysmal activity of high frequency (>5 Hz) characterized by a >3-fold amplitude increment over baseline with progression of the spike frequency that lasted for a minimum of 3 s^33^,^50^. Seizure severity was scored using the scale of Racine^51^: 1, chewing or mouth and facial movements; 2, head nodding; 3, forelimb clonus; 4, generalized seizure with rearing; 5, generalized seizure with rearing and falling. In the chronic phase, animals were continuously video-EEG recorded for a week after the first spontaneous seizure, for a week before the sacrifice and 48 h per week between the first spontaneous seizure and the killing.

### Tissue preparation

Rats were killed by decapitation under an anesthetic overdose. Before being killed, rats were anesthetized with ketamine (870 mg/kg) and xylazine (13 mg/Kg), and a blood sample was collected through intracardiac withdrawal. Blood samples were added EDTA (ethylenediaminetetraacetic acid disodium salt dehydrate, Sigma, Germany) to prevent clotting and plasma was separated by centrifugation at 1000 rcf for 2 minutes and the supernatant was centrifuged at 2500 rcf for 2 minutes. This procedure ensured low platelet contamination^52^. Samples were separated in 200 μl aliquots and stored at −80 °C.

Brains were rapidly removed, frozen in isopentane and stored at –80 °C until use. Coronal sections (20 μm thick) were cut across the entire hippocampus, plates 40–52 of Pellegrino *et al.*^47^ and mounted onto slides for laser microdissection (MembraneSlides PEN-Membrane 2,0 μm, Leica). Sections were fixed according to a slight modification of a published protocol^53^: ethanol 70% in diethyl pyrocarbonate (DEPC) water for 5 min, rinse in distilled DEPC water, incubation in Mayer’s hematoxylin solution 0.1% (Sigma) for 5 min, another wash in DEPC water; finally, section were dried at room temperature for 10 min before being stored at –80 °C. This procedure ensured the best yield and quality of extracted RNA, as compared to other published procedures, in side-by-side testing experiments conducted in the lab before initiating the processing of the tissue employed in the present study.

### Human Samples

All human hippocampal tissue was from autopsies of 2 epileptic and 10 non-epileptic patients who died of acute lung or heart pathologies. All autoptic material was from the tissue archives of the Bellaria Hospital (Bologna, Italy). The study was approved by the Ethics Committee of Bologna (Comitato Etico Indipendente dell’Azienda USL della Città di Bologna). Since this was a pilot study in a small cohort of cases, all from archive samples of patients who died years ago, the Ethical Committee acknowledged the impossibility to collect informed consents and approved the study under the condition to strictly ensure anonymity. All the methods were carried out in accordance with the approved guidelines, and all information regarding the human material was managed using anonymous numerical codes and samples were handled in compliance with the Helsinki declaration (http://www.wma.net/en/30publications/10policies/b3/). Specimens were formalin-fixed and paraffin-embedded. They were de-waxed using Bio-Clear (Bio-Optica, Milan, Italy), washed in ethanol and stained with hematoxylin and eosin, as indicated in Zucchini *et al.*^19^.

### Laser microdissection, RNA purification and profiling

**Figure 6:**
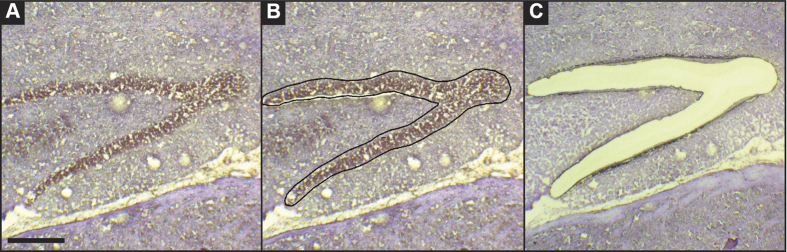
Laser microdissection of the rat granule cell layer. Representative hematoxylin stained hippocampal section prepared from a pilocarpine treated rat (level: dentate gyrus of the hippocampus). (**A**) Intact section before laser microdissection. (**B**) Identification of the region of interest, the granular layer (black line). (**C**) The section after the laser microdissection, without the granular layer. Horizontal bar = 320 μm.

Ten-micron-thick sections were cut using a microtome and collected in slides for laser microdissection (LMD). LMD was performed essentially as previously described^19^,^54^,^55^. Briefly, PEN-membrane slides were mounted on a Leica LMD6000B system (Leica Microsystem, Wetzlar, Germany) with the sections facing downwards. The intensity, aperture and cutting velocity were calibrated to obtain the sharpest cut with the minimal intensity, then the pulsed UV laser beam was carefully directed along the borders of the dentate gyrus GCL (Fig. 6). The microdissected region was then transferred by gravity into a 0,2 ml Eppendorf tube cap placed directly underneath the tissue section. Tissue collection was verified by inspecting the tube cap. Granule cells were collected from at least 9 slices per animal and 3–4 slices per patient, in order to obtain an adequate amount of tissue for RNA extraction. Material from all sections from the same animal or patient was pooled together, and total RNA purified using an RNA purification kit (miRNeasy Micro kit from QIAGEN Germany, was used to purify the RNA in animal samples and RecoverAll™ Total Nucleic Acid Isolation kit from Life Technologies, Milan, Italy for human samples). Approximately 250 ng of total RNA were obtained from each sample. Every RNA sample was analyzed using an Agilent 2100 Bioanalyzer in order to evaluate the quality of total RNA. RNA samples with a RNA Integrity Number (RIN) <5 (in a 1 to 10 scale) were excluded from further analysis.

Total RNA, including microRNAs , was also purified from 200 μl of plasma samples with a specific RNA purification kit (miRNeasy Serum/Plasma kit, Qiagen). To ensure effective denaturation of proteins, 10 volumes of Qiazol solution were added to one volume of plasma or serum. Samples were then thoroughly mixed by vortexing and incubated at room temperature for 5 min^56^. As internal control, we employed *c. elegans* miR-39 miRNA mimic (miRNeasy Serum/Plasma Spike-In Control, Qiagen; 3.5 μl, 1.6 × 108 copies/μl). Total RNA was eluted from column by two sequential elutions with 16 μl of RNase-free water to yield 30 μl RNA solution. Prior to RNA isolation, all samples were carefully ordered to alternate samples from different groups, in order to avoid biases related to batch effects and geographic location of samples in the thermocycler block^56^.

Total RNA was used for microarray analysis (Rat miRNA MicroArray Kit, Human micro-RNA Microarray V3, #G4470C, Agilent Technologies, Santa Clara, CA, USA). The chip consisted of 60-mer DNA probes and allowed simultaneous analysis of 677 rat miRNAs and 1200 human miRNAs obtained from the Sanger miR-BASE database (Release 16.0 for rats and 10.1 for humans). We employed approximately 100 ng total RNA per sample in each experiment. RNA labeling and hybridization were performed according to the manufacturer’s indications. Agilent scanner and the Feature Extraction 10.5 software (Agilent Technologies) were used to obtain the raw-data.

### Data analysis

Microarray results were analyzed by using the GeneSpring GX 13 software (Agilent Technologies). Data transformation was applied to set all the negative raw values at 1.0, followed by quantile normalization. Filters on gene expression were used to keep only those miRNAs that were detected in at least one sample. Comparisons among all experimental groups (controls, early latency, late latency, 12 h within the first seizure and chronic) were performed with ANOVA analysis and differences were considered significant when adjusted P was <0.1. Comparisons between two groups were performed with moderated t-test and considering an adjusted p < 0.1 as significant. For plasma, we applied ANOVA analysis and a P cutoff of 0.05. Gaussian graphical models (GGM) based on partial correlation were constructed using a FDR threshold of 1%. Principle components analysis (PCA) and boxplots were generated in R. Hierarchical clustering was performed on differentially expressed miRNAs with GeneSpring GX 12 software (Agilent Technologies), using the Pearson correlation as a measure of similarity. For cluster image generation, expression data was centered on gene median across samples.

Predicted target genes for each miRNA that was deregulated during the two earlier time point (4 and 7 days after SE) were obtained from miRWalk using the mirDB and TargetScan algorithms^57^. Only those genes that were predicted by both algorithms were taken in consideration. The list of targets was further filtered using publicly available rat gene expression from the dentate gyrus (GSE49850). The predicted targets were filtered out if they were not present in the top two-thirds of expressed genes assayed in the control rats. Gene ontology (GO) and Kyoto encyclopedia of genes and genomes (KEGG) enrichment was performed using webgestalt (http://bioinfo.vanderbilt.edu/webgestalt/) and the terms enriched with adjusted p-values < 0.05 were deemed significant.

### RT-qPCR

Reverse transcription (RT) reactions were performed using the Taq-Man miRNA Reverse Transcription kit and miRNA-specific stem-loop primers (Applied Biosystems, Inc) in a scaled down (7.5 μl) reaction. Samples were run at 16 °C for 30 min, 42 °C for 30 min, 85 °C for 5 min and hold at 4 °C. RT products were stored undiluted at −20 °C prior to running real-time PCR.

For GCL samples, real-time PCR reactions were performed using the TaqMan miRNA assay kit (Applied Biosystems, Santa Clara, CA, USA) according to the manufacturer’s instructions; for plasma, they were performed as described by others^56^. Samples were run in triplicate at 95 °C for 15 sec and 60 °C for 1 min using CFX96 Touch™ (Biorad, Milan, Italy). Analysis was performed by the comparative delta threshold cycle (ΔCT) method^58^. For brain samples, U6 and snoRNA were used as reference genes^59^. For plasma, data normalization was obtained using the synthetic spiked-in *c. elegans* miR-39.

## Additional Information

**How to cite this article**: Roncon, P. *et al.* MicroRNA profiles in hippocampal granule cells and plasma of rats with pilocarpine-induced epilepsy - comparison with human epileptic samples. *Sci. Rep.* **5**, 14143; doi: 10.1038/srep14143 (2015).

## Supplementary Material

Supplementary Information

## Figures and Tables

**Table 1:** miRNAs expression patterns in chronically epileptic rats and patients.

	**Chronic rats**		**Patients**	
	**Fold change**	**Regulation**	**Fold change**	**Regulation**
miR-21-5p	1.24	Up	1.93	Up
miR-23a- 5p	1.83	Up	3.35	Up
miR-146a-5p	21.24	Up	3.63	Up
miR-181c-5p	1.91	Up	1.88	Up

## References

[b1] Simonato M. *et al.* The challenge and promise of anti-epileptic therapy development in animal models. Lancet Neurol 13, 949–60 (2014). 25127174 10.1016/S1474-4422(14)70076-6 PMC5003536

[b2] Simonato M. *et al.* Finding a better drug for epilepsy: preclinical screening strategies and experimental trial design. Epilepsia 53, 1860–7 (2012). 22708847 10.1111/j.1528-1167.2012.03541.x PMC4208688

[b3] Simonato M., French J. A., Galanopoulou A. S. & O’Brien T. J. Issues for new antiepilepsy drug development. Curr Opin Neurol 26, 195–200 (2013). 23406913 10.1097/WCO.0b013e32835efe29 PMC3770466

[b4] Galanopoulou A. S. *et al.* Identification of new epilepsy treatments: issues in preclinical methodology. Epilepsia 53, 571–82 (2012). 22292566 10.1111/j.1528-1167.2011.03391.x PMC3551973

[b5] Engel J. Jr. *et al.* Epilepsy biomarkers. Epilepsia 4, 61–9 (2013). 23909854 10.1111/epi.12299 PMC4131763

[b6] Bartel D. P. MicroRNAs: genomics, biogenesis, mechanism, and function. Cell 116, 281–97 (2004). 14744438 10.1016/s0092-8674(04)00045-5

[b7] Im H. I. & Kenny P. J. MicroRNAs in neuronal function and dysfunction. Trends Neurosci 35, 325–34 (2012). 22436491 10.1016/j.tins.2012.01.004 PMC3565236

[b8] McNeill E. & Van Vactor D. MicroRNAs shape the neuronal landscape. Neuron 75, 363–79 (2012). 22884321 10.1016/j.neuron.2012.07.005 PMC3441179

[b9] Vezzani A., Aronica E., Mazarati A. & Pittman Q. J. Epilepsy and brain inflammation. Exp Neurol 244, 11–21 (2013). 21985866 10.1016/j.expneurol.2011.09.033

[b10] Mikkonen M. *et al.* Remodeling of neuronal circuitries in human temporal lobe epilepsy: increased expression of highly polysialylated neural cell adhesion molecule in the hippocampus and the entorhinal cortex. Ann Neurol 44, 923–34 (1998). 9851437 10.1002/ana.410440611

[b11] Blümcke I. *et al.* International consensus classification of hippocampal sclerosis in temporal lobe epilepsy: a Task Force report from the ILAE Commission on Diagnostic Methods. Epilepsia 54, 1315–29 (2013). 23692496 10.1111/epi.12220

[b12] Wu J. *et al.* MicroRNA-421 is a new potential diagnosis biomarker with higher sensitivity and specificity than carcinoembryonic antigen and cancer antigen 125 in gastric cancer. Biomarkers 16, 1–6 (2014). 10.3109/1354750X.2014.992812 25510566

[b13] Sepramanian S. *et al.* Circulating microRNA as biomarkers of acute stroke. Int J Mol Sci 15, 1418–32 (2014). 24447930 10.3390/ijms15011418 PMC3907877

[b14] Tan L. *et al.* Genome-wide serum microRNA expression profiling identifies serum biomarkers for Alzheimer’s disease. J Alzheimers Dis 40, 1017–27 (2014). 24577456 10.3233/JAD-132144

[b15] Cloutier F., Marrero A., O’Connell C. & Morin P. J. MicroRNAs as Potential Circulating Biomarkers for Amyotrophic Lateral Sclerosis. J Mol Neurosci 56, 102–112 (2014). 25433762 10.1007/s12031-014-0471-8

[b16] Jin X. F., Wu N., Wang L. & Li J. Circulating microRNAs: a novel class of potential biomarkers for diagnosing and prognosing central nervous system diseases. Cell Mol Neurobiol 33, 601–13 (2013). 23633081 10.1007/s10571-013-9940-9 PMC11497935

[b17] Zhang Y. *et al.* Altered expression levels of miRNAs in serum as sensitive biomarkers for early diagnosis of traumatic injury. J Cell Biochem 112, 2435–42 (2011). 21538484 10.1002/jcb.23168

[b18] Kan A. A. *et al.* Genome-wide microRNA profiling of human temporal lobe epilepsy identifies modulators of the immune response. Cell Mol Life Sci 69, 3127–45 (2012). 22535415 10.1007/s00018-012-0992-7 PMC3428527

[b19] Zucchini S. *et al.* Identification of miRNAs differentially expressed in human epilepsy with or without granule cell pathology. PLoS One 9, e105521. 10.1371/journal.pone.0105521 (2014). 25148080 PMC4141756

[b20] Risbud R. M. & Porter B. E. Changes in microRNA expression in the whole hippocampus and hippocampal synaptoneurosome fraction following pilocarpine induced status epilepticus. PLoS One 8, e53464. 10.1371/journal.pone.0053464 (2013). 23308228 PMC3538591

[b21] Hu K. *et al.* MicroRNA expression profile of the hippocampus in a rat model of temporal lobe epilepsy and miR-34a-targeted neuroprotection against hippocampal neurone cell apoptosis post-status epilepticus. BMC Neurosci 13, 115 (2012). 22998082 10.1186/1471-2202-13-115 PMC3471047

[b22] Jimenez-Mateos E. M. *et al.* miRNA Expression profile after status epilepticus and hippocampal neuroprotection by targeting miR-132. Am J Pathol 179, 2519–32 (2011). 21945804 10.1016/j.ajpath.2011.07.036 PMC3204080

[b23] Bot A. M., Dębski K. J. & Lukasiuk K. Alterations in miRNA levels in the dentate gyrus in epileptic rats. PLoS One 8, e76051. 10.1371/journal.pone.0076051 (2013). 24146813 PMC3795667

[b24] Gorter J. A. *et al.* Hippocampal subregion-specific microRNA expression during epileptogenesis in experimental temporal lobe epilepsy. Neurobiol Dis 62, 508–20 (2013). 24184920 10.1016/j.nbd.2013.10.026

[b25] Lucherini O. M. *et al.* First report of circulating microRNAs in tumour necrosis factor receptor-associated periodic syndrome (TRAPS). PLoS One 8, e73443. 10.1371/journal.pone.0073443 (2013). 24066048 PMC3774691

[b26] Meng F. *et al.* Neuronal calcium signaling pathways are associated with the development of epilepsy. Mol Med Rep 11, 196–202 (2015). 25339366 10.3892/mmr.2014.2756 PMC4237086

[b27] Yelamanchili S. V. & Fox H. S. Defining larger roles for “tiny” RNA molecules: role of miRNAs in neurodegeneration research. J Neuroimmune Pharmacol 5, 63–9 (2010). 19757077 10.1007/s11481-009-9172-4 PMC2881294

[b28] Bienvenu T., Diebold B., Chelly J. & Isidor B. Refining the phenotype associated with MEF2C point mutations. Neurogenetics 14, 71–5 (2013). 23001426 10.1007/s10048-012-0344-7

[b29] Johnson M. R. *et al.* Systems genetics identifies Sestrin 3 as a regulator of a proconvulsant gene network in human epileptic hippocampus. Nat Commun 6, 6031 (2015). 25615886 10.1038/ncomms7031 PMC4627576

[b30] Iyer A. *et al.* MicroRNA-146a: a key regulator of astrocyte-mediated inflammatory response. PLoS One 7, e44789. 10.1371/journal.pone.0044789 (2012). PMC3441440 23028621

[b31] Pitkänen A. & Sutula T. P. Is epilepsy a progressive disorder? Prospects for new therapeutic approaches in temporal-lobe epilepsy. Lancet Neurol 1, 173–81 (2002). 12849486 10.1016/s1474-4422(02)00073-x

[b32] Curia G., Longo D., Biagini G., Jones R. S. & Avoli M. The pilocarpine model of temporal lobe epilepsy. J Neurosci Methods 172, 143–57 (2008). 18550176 10.1016/j.jneumeth.2008.04.019 PMC2518220

[b33] Paradiso B. *et al.* Localized overexpression of FGF-2 and BDNF in hippocampus reduced mossy fibers sprouting and spontaneous seizures up to 4 weeks after pilocarpine-induced status epilepticus. Epilepsia 52, 572–78 (2011). 21269288 10.1111/j.1528-1167.2010.02930.x

[b34] Wang X. *et al.* M129V polymorphism in the prion protein gene is not associated with mesial temporal lobe epilepsy in a Han Chinese population. Eur J Neurol 15, 827–30 (2008). 18549399 10.1111/j.1468-1331.2008.02191.x

[b35] Schauwecker P. E. Galanin receptor 1 deletion exacerbates hippocampal neuronal loss after systemic kainate administration in mice. PLoS One 5, e15657. 10.1371/journal.pone.0015657 (2010). 21179451 PMC3001489

[b36] McColl C. D., Jacoby A. S., Shine J., Iismaa T. P. & Bekkers J. M. Galanin receptor-1 knockout mice exhibit spontaneous epilepsy, abnormal EEGs and altered inhibition in the hippocampus. Neuropharmacology 50, 209–18 (2006). 16243364 10.1016/j.neuropharm.2005.09.001

[b37] Szczurowska E. & Mareš P. NMDA and AMPA receptors: development and status epilepticus. Physiol Res 62, S21–38 (2013). 24329701 10.33549/physiolres.932662

[b38] Pitkänen A. *et al.* Issues related to development of antiepileptogenic therapies. Epilepsia 4, 35–43 (2013). 23909852 10.1111/epi.12297 PMC3740390

[b39] Aronica E. *et al.* Expression pattern of miR-146a, an inflammation-associated microRNA, in experimental and human temporal lobe epilepsy. Eur J Neurosci 31, 1100–7 (2010). 20214679 10.1111/j.1460-9568.2010.07122.x

[b40] Song Y. J. *et al.* Temporal lobe epilepsy induces differential expression of hippocampal miRNAs including let-7e and miR-23a/b. Brain Res 1387, 134–40 (2011). 21376023 10.1016/j.brainres.2011.02.073

[b41] Cheng A. M., Byrom M. V., Shelton J. & Ford L. P. Antisense inhibition of human miRNAs and indications for an involvement of miRNA in cell growth and apoptosis. Nucleic Acids Res 33, 1290–7 (2005). 15741182 10.1093/nar/gki200 PMC552951

[b42] Song Y. J. *et al.* miR-23 regulation of lamin B1 is crucial for oligodendrocyte development and myelination. Dis Model Mech 2, 178–88 (2009). 19259393 10.1242/dmm.001065 PMC2650193

[b43] Valadi H. *et al.* Exosome-mediated transfer of mRNAs and microRNAs is a novel mechanism of genetic exchange between cells. Nat Cell Biol 9, 654–9 (2007). 17486113 10.1038/ncb1596

[b44] Hunter M. P. *et al.* Detection of microRNA expression in human peripheral blood microvesicles. PLoS One 3, e3694. 10.1371/journal.pone.0003694 (2008). 19002258 PMC2577891

[b45] Liu D. Z. *et al.* Brain and blood microRNA expression profiling of ischemic stroke, intracerebral hemorrhage, and kainate seizures. J Cereb Blood Flow Metab 30, 92–101 (2010). 19724284 10.1038/jcbfm.2009.186 PMC2949089

[b46] Kilkenny C. *et al.* Animal research: reporting *in vivo* experiments: the ARRIVE guidelines. J Cereb Blood Flow Metab 31, 991–3 (2011). 21206507 10.1038/jcbfm.2010.220 PMC3070981

[b47] Pellegrino L. J., Pellegrino A. S. & Cushman A. J. A stereotaxic atlas of the rat brain. Plenum Press, New York and London (1979).

[b48] Paradiso B. *et al.* Localized delivery of fibroblast growth factor-2 and brain-derived neurotrophic factor reduces spontaneous seizures in an epilepsy model. Proc Natl Acad Sci USA 106, 7191–6 (2009). 19366663 10.1073/pnas.0810710106 PMC2678472

[b49] Mazzuferi M. *et al.* Enhancement of GABA(A)-current run-down in the hippocampus occurs at the first spontaneous seizure in a model of temporal lobe epilepsy. Proc Natl Acad Sci USA 107, 3180–5 (2010). 20133704 10.1073/pnas.0914710107 PMC2840298

[b50] Soukupova M. *et al.* Impairment of GABA release in the hippocampus at the time of the first spontaneous seizure in the pilocarpine model of temporal lobe epilepsy. Exp Neurol 257, 39–49 (2014). 24768627 10.1016/j.expneurol.2014.04.014

[b51] Racine R. J. Modification of seizure activity by electrical stimulation. II. Motor seizure. Electroencephalogr Clin Neurophysiol 32, 281–94 (1972). 4110397 10.1016/0013-4694(72)90177-0

[b52] Cheng H. H. *et al.* Plasma processing conditions substantially influence circulating microRNA biomarkers levels. PLoS One 8, e64795. 10.1371/journal.pone.0064795 (2013). 23762257 PMC3676411

[b53] Goldsworthy S. M., Stocktown P. S., Trempus C. S., Foley J. F. & Maronpot R. R. Effects of fixation on RNA extraction and amplification from laser capture microdissected tissue. Mol Carcinog 25, 86–91 (1999). 10365909

[b54] Burbach G. J., Dehn D., Del Turco D. & Deher T. Quantification of layer-specific gene expression in the hippocampus: effective use of laser microdissection in combination with quantitative RT-PCR. J of Neurosci Methods 131, 83–91(2003). 14659827 10.1016/s0165-0270(03)00232-2

[b55] Baj G. *et al.* Regulation of the spatial code for BDNF mRNA isoform in the rat hippocampus following pilocarpine-treatment: a systematic analysis using laser microdissection and quantitative real-time PCR. Hippocampus 23, 413–23 (2013). 23436435 10.1002/hipo.22100

[b56] Kroh E. M., Parkin R. K., Mitchell P. S. & Tewari M. Analysis of circulating microRNA biomarkers in plasma and serum using quantitative reverse transcription-PCR (qRT-PCR). Methods 50, 298–301 (2010). 20146939 10.1016/j.ymeth.2010.01.032 PMC4186708

[b57] Dweep H., Sticht C., Pandey P. & Gretz N. miRWalk-database: prediction of possible miRNA binding sites by “walking” the genes of 3 genomes. J Neurosci Methods 208, 44–7 (2011). 10.1016/j.jbi.2011.05.002 21605702

[b58] Livak K. J. & Schmittgen T. D. Analysis of relative gene expression data using real-time quantitative PCR and the 2^−ΔΔCT^ method. Methods 25, 402–8 (2001). 11846609 10.1006/meth.2001.1262

[b59] de Araújo M. A. *et al.* Identification of endogenous reference genes for the analysis of microRNA expression in the hippocampus of the pilocarpine-induced model of mesial temporal lobe epilepsy. PLoS One 9, e100529. 10.1371/journal.pone.0100529 (2014). 24964029 PMC4070922

