# Supplementary material for: MicroRNA profiles in hippocampal granule cells and plasma of rats with pilocarpine-induced epilepsy – comparison with human epileptic samples
Source: Sci Rep. 2015 Sep 18;5:14143. doi: 10.1038/srep14143 (PMC4585664; doi:10.1038/srep14143)
Supplement: Supplementary Information [file srep14143-s1.pdf]

Supplementary information

MicroRNA profiles in hippocampal granule cells and plasma of rats with pilocarpine-induced epilepsy – comparison with human epileptic samples.

Paolo Roncon, Marie Soukupovà, Anna Binaschi, Chiara Falcicchia, Silvia Zucchini, Manuela Ferracin, Sarah R Langley, Enrico Petretto, Michael R Johnson, Gianluca Marucci, Roberto Michelucci, Guido Rubboli, Michele Simonato

Contents

- Supplementary Tables S1-S5
- Supplementary Figures S1-S7

Supplementary Table S1. Significantly dys-regulated miRNAs in the GCL of epileptic compared with control animals.

miRNA	P	P adjusted
**miR-21-5p**	$6,57^{-09}$	$1,73^{-06}$
**miR-132-3p**	$1,36^{-07}$	$1,19^{-05}$
**miR-20a-5p**	$1,35^{-07}$	$1,19^{-05}$
**miR-212-3p**	$5,71^{-07}$	$3,75^{-05}$
**miR-29c-5p**	$8,57^{-07}$	$4,5^{-05}$
**miR-652-3p**	$4,55^{-06}$	$1,99^{-04}$
**miR-551b-3p**	$1,31^{-05}$	$4,91^{-04}$
**miR-92a-3p**	$1,64^{-05}$	$5,38^{-04}$
**miR-344b-2-3p**	$2,16^{-05}$	$6,32^{-04}$
**miR-15b-5p**	$5,61^{-05}$	$1,46^{-03}$
**miR-24-3p**	$6,12^{-05}$	$1,46^{-03}$
**miR-23a-3p**	0,0001	$2,44^{-03}$
**miR-222-3p**	0,0002	$3,23^{-03}$
**miR-181d-5p**	0,0002	$3,97^{-03}$
**miR-674-3p**	0,0002	$4,28^{-03}$
**miR-142-3p**	0,0003	$4,96^{-03}$
**miR-17-5p**	0,0003	$4,96^{-03}$
**miR-874-3p**	0,0004	$6,00^{-03}$
**miR-27a-3p**	0,0004	$6,00^{-03}$
**miR-19b-3p**	0,001	$1,37^{-02}$
**miR-93-5p**	0,0011	$1,39^{-02}$
**miR-433-3p**	0,0012	$1,45^{-02}$
**miR-30a-5p**	0,0013	$1,47^{-02}$
**miR-18a-5p**	0,0016	$1,53^{-02}$
**miR-146a-5p**	0,0015	$1,53^{-02}$
**miR-20b-5p**	0,0014	$1,53^{-02}$
**miR-138-5p**	0,0015	$1,53^{-02}$
**miR-431**	0,0024	$2,21^{-02}$
**miR-130a-3p**	0,0024	$2,21^{-02}$
**miR-7a-5p**	0,0025	$2,22^{-02}$
**miR-3584-5p**	0,0028	$2,41^{-02}$
**miR-23b-3p**	0,0035	$2,87^{-02}$
**miR-181c-5p**	0,0051	$4,14^{-02}$
**miR-30e-3p**	0,0056	$4,34^{-02}$
**miR-129-5p**	0,0062	$4,67^{-02}$
**miR-296-5p**	0,0065	$4,72^{-02}$
**miR-505-3p**	0,0073	$5,05^{-02}$
**miR-19a-3p**	0,0073	$5,05^{-02}$

**miR-154-3p**	0,0077	$5,19^{-02}$
**miR-139-5p**	0,0084	$5,51^{-02}$
**miR-204-5p**	0,009	$5,81^{-02}$
**miR-410-3p**	0,0096	$6,04^{-02}$
**miR-129-1-3p**	0,0100	$6,13^{-02}$
**miR-186-5p**	0,0107	$6,40^{-02}$
**miR-337-5p**	0,0114	$6,48^{-02}$
**miR-324-3p**	0,0111	$6,48^{-02}$
**miR-485-5p**	0,0115	$6,48^{-02}$
**miR-466b-5p**	0,0124	$6,81^{-02}$
**let-7c-5p**	0,0127	$6,85^{-02}$
**miR-873-5p**	0,0142	$7,50^{-02}$
**miR-344b-5p**	0,0154	$7,89^{-02}$
**miR-380-3p**	0,0157	$7,89^{-02}$
**let-7b-5p**	0,0162	$7,89^{-02}$
**miR-107-3p**	0,0162	$7,89^{-02}$
**miR-140-3p**	0,0174	$8,33^{-02}$
**miR-409a-5p**	0,0182	$8,56^{-02}$
**miR-7a-1-3p**	0,0186	$8,60^{-02}$
**miR-30c-2-3p**	0,0195	$8,70^{-02}$
**miR-129-2-3p**	0,0192	$8,70^{-02}$
**miR-500-3p**	0,0202	$8,87^{-02}$
**miR-212-5p**	0,0217	$9,37^{-02}$
**miR-328a-3p**	0,0222	$9,43^{-02}$
**miR-665**	0,0231	$9,64^{-02}$

Supplementary Table S2. Significantly dys-regulated miRNAs in the GCL in latency (four and eight days after SE) compared with control animals.

	P	Fold change	Regulation
**miR-142-3p**	0,067	34,11	up
**miR-344b-2-3p**	0,030	23,32	up
**miR-3584-5p**	0,089	18,28	up
**miR-17-5p**	0,047	18,16	up
**miR-30c-2-3p**	0,077	11,94	down
**miR-181a-1-3p**	0,093	9,02	down
**miR-132-3p**	0,002	4,58	up
**miR-21-5p**	0,008	3,56	up
**miR-212-3p**	0,002	2,95	up
**miR-181d-5p**	0,022	2,57	up
**miR-27a-3p**	0,026	2,08	up
**miR-30e-3p**	0,030	2,06	down
**miR-92a-3p**	0,046	2,04	up
**miR-20a-5p**	0,030	2,00	up
**miR-874-3p**	0,093	2,00	up
**miR-139-5p**	0,094	1,97	down
**miR-431**	0,091	1,88	up
**miR-30a-3p**	0,094	1,82	down
**miR-551b-3p**	0,030	1,71	down
**miR-23a-3p**	0,070	1,65	up
**miR-433-3p**	0,033	1,59	up
**miR-330-3p**	0,091	1,58	down
**miR-370-3p**	0,071	1,53	up
**miR-138-5p**	0,022	1,50	down
**miR-140-3p**	0,030	1,48	down
**miR-128-3p**	0,053	1,43	down
**miR-380-3p**	0,041	1,43	up
**miR-29c-5p**	0,008	1,43	down
**miR-30a-5p**	0,030	1,39	down
**miR-300-3p**	0,077	1,30	up
**miR-652-3p**	0,070	1,29	down
**miR-186-5p**	0,100	1,24	down
**miR-103-3p**	0,070	1,11	down

Supplementary Table S3. Significantly dys-regulated miRNAs in the GCL of rats at the time of the first spontaneous seizure compared with controls.

	P	Fold change	Regulation
**miR-142-3p**	0,067	34,11	up
**miR-344b-2-3p**	0,030	23,32	up
**miR-3584-5p**	0,089	18,28	up
**miR-17-5p**	0,047	18,16	up
**miR-30c-2-3p**	0,077	11,94	down
**miR-181a-1-3p**	0,093	9,02	down
**miR-132-3p**	0,002	4,58	up
**miR-21-5p**	0,008	3,56	up
**miR-212-3p**	0,002	2,95	up
**miR-181d-5p**	0,022	2,57	up
**miR-27a-3p**	0,026	2,08	up
**miR-30e-3p**	0,030	2,06	down
**miR-92a-3p**	0,046	2,04	up
**miR-20a-5p**	0,030	2,00	up
**miR-874-3p**	0,093	2,00	up
**miR-139-5p**	0,094	1,97	down
**miR-431**	0,091	1,88	up
**miR-30a-3p**	0,094	1,82	down
**miR-551b-3p**	0,030	1,71	down
**miR-23a-3p**	0,070	1,65	up
**miR-433-3p**	0,033	1,59	up
**miR-330-3p**	0,091	1,58	down
**miR-370-3p**	0,071	1,53	up
**miR-138-5p**	0,022	1,50	down
**miR-140-3p**	0,030	1,48	down
**miR-128-3p**	0,053	1,43	down
**miR-380-3p**	0,041	1,43	up
**miR-29c-5p**	0,008	1,43	down
**miR-30a-5p**	0,030	1,39	down
**miR-300-3p**	0,077	1,30	up
**miR-652-3p**	0,070	1,29	down
**miR-186-5p**	0,100	1,24	down
**miR-103-3p**	0,070	1,11	down

Supplementary Table S4. Significantly dys-regulated miRNAs in the GCL of chronically epileptic compared with control animals.

	P	Fold change	Regulation
**miR-154-3p**	$8{,}608^{-04}$	36,68	up
**miR-146a-5p**	$4{,}274^{-02}$	21,24	up
**miR-344b-2-3p**	$8{,}079^{-02}$	18,01	up
**miR-31a-5p**	$4{,}962^{-02}$	4,53	down
**miR-181d-5p**	$2{,}018^{-02}$	3,62	up
**miR-132-3p**	$2{,}018^{-02}$	3,51	up
**miR-551b-3p**	$2{,}357^{-02}$	2,75	down
**miR-212-3p**	$2{,}018^{-02}$	2,39	up
**miR-873-5p**	$2{,}768^{-02}$	2,28	down
**miR-652-3p**	$2{,}018^{-02}$	1,93	down
**miR-181c-5p**	$5{,}467^{-02}$	1,91	up
**miR-23a-3p**	$2{,}018^{-02}$	1,84	up
**miR-433-5p**	$2{,}357^{-02}$	1,71	up
**miR-433-3p**	$5{,}082^{-02}$	1,70	up
**miR-409a-5p**	$8{,}079^{-02}$	1,60	up
**miR-410-3p**	$8{,}079^{-02}$	1,53	up
**miR-1949**	$4{,}274^{-02}$	1,48	down
**miR-380-3p**	$8{,}079^{-02}$	1,45	up
**miR-30a-5p**	$2{,}392^{-02}$	1,45	down
**miR-411-3p**	$8{,}079^{-02}$	1,40	up
**miR-140-3p**	$8{,}079^{-02}$	1,32	down
**miR-664-3p**	$8{,}079^{-02}$	1,31	down
**miR-29c-5p**	$2{,}018^{-02}$	1,28	down
**miR-21-5p**	$5{,}214^{-02}$	1,25	up
**miR-148b-3p**	$8{,}079^{-02}$	1,23	down

Supplementary Table S5. Significantly dys-regulated miRNAs in the plasma of epileptic compared with control animals.

miRNA	P	P adjusted
**miR.598.5p**	0,0002	0,0833
**miR.300.3p**	0,0160	0,4286
**miR.381.3p**	0,0069	0,4286
**miR.3582**	0,0027	0,4286
**miR.199a.5p**	0,0076	0,4286
**miR.181a.1.3p**	0,0131	0,4286
**miR.30c.2.3p**	0,0103	0,4286
**miR.10b.5p**	0,0164	0,4286
**miR.214.3p**	0,0097	0,4286
**miR.466b.1.3p**	0,0099	0,4286
**miR.144.3p**	0,0141	0,4286
**miR.142.3p**	0,0154	0,4286
**miR.3065.3p**	0,0074	0,4286
**miR.125b.2.3p**	0,0263	0,4853
**miR.466c.3p**	0,0300	0,4853
**miR.429**	0,0326	0,4853
**miR.32.3p**	0,0329	0,4853
**miR.374.5p**	0,0234	0,4853
**miR.208a.5p**	0,0323	0,4853
**miR.328a.3p**	0,0278	0,4853
**miR.210.3p**	0,0328	0,4853
**miR.494.3p**	0,0231	0,4853
**miR.465.5p**	0,0324	0,4853
**miR.142.5p**	0,0375	0,5293
**miR.1188.3p**	0,0395	0,5353
**miR.770.3p**	0,0467	0,5676
**miR.101b.3p**	0,0486	0,5676
**miR.598.5p**	0,0477	0,5676
**miR.300.3p**	0,0160	0,4286

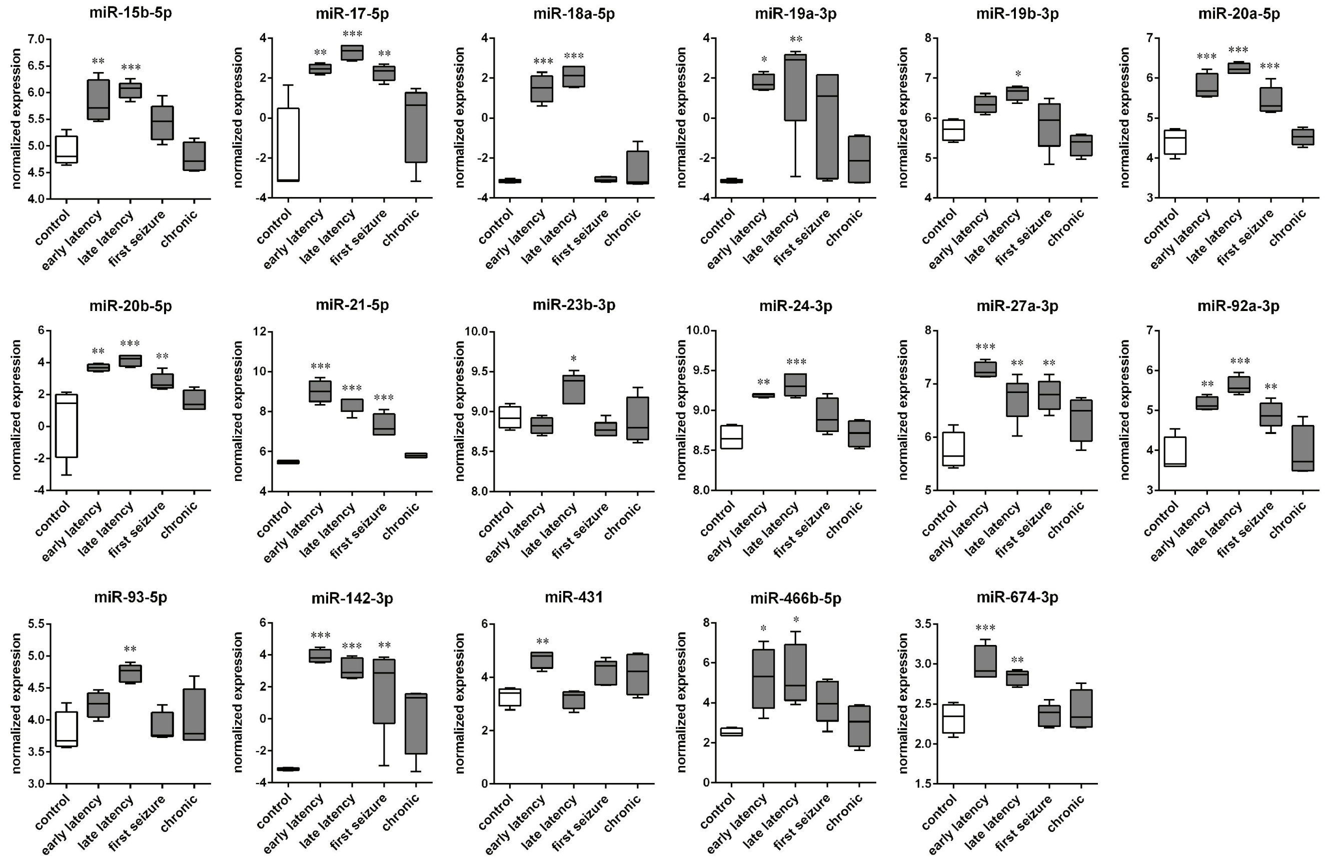

Supplementary Fig. S1. Up-regulated miRNAs in the rat granule cell layer (GCL) during latency. The boxplots depict the time course of expression for 17 of the miRNAs identified as significant with a false discovery rate (FDR)<10% in the GCL by using one-way ANOVA. Each boxplot represents 4 animals. * $p<0.05$; ** $p < 0.01$; *** $p<0.001$; Tukey's test.

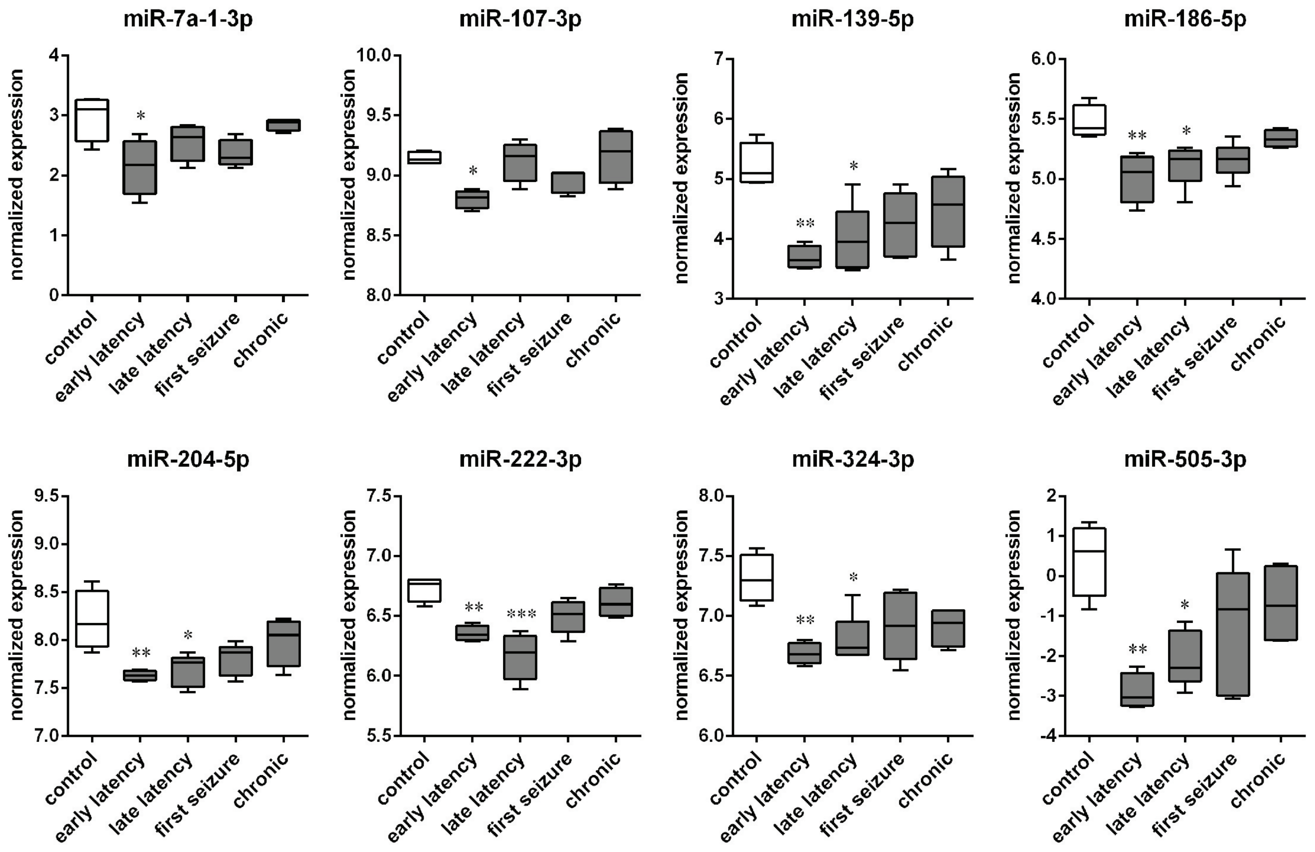

Supplementary Fig. S2. Down-regulated miRNAs in the rat granule cell layer (GCL) during latency. The boxplots depict the time course of expression for 8 of the miRNAs identified as significant with a false discovery rate (FDR)<10% in the GCL by using one-way ANOVA. Each boxplot represents 4 animals. * $p<0.05$; ** $p < 0.01$; *** $p<0.001$; Tukey's test.

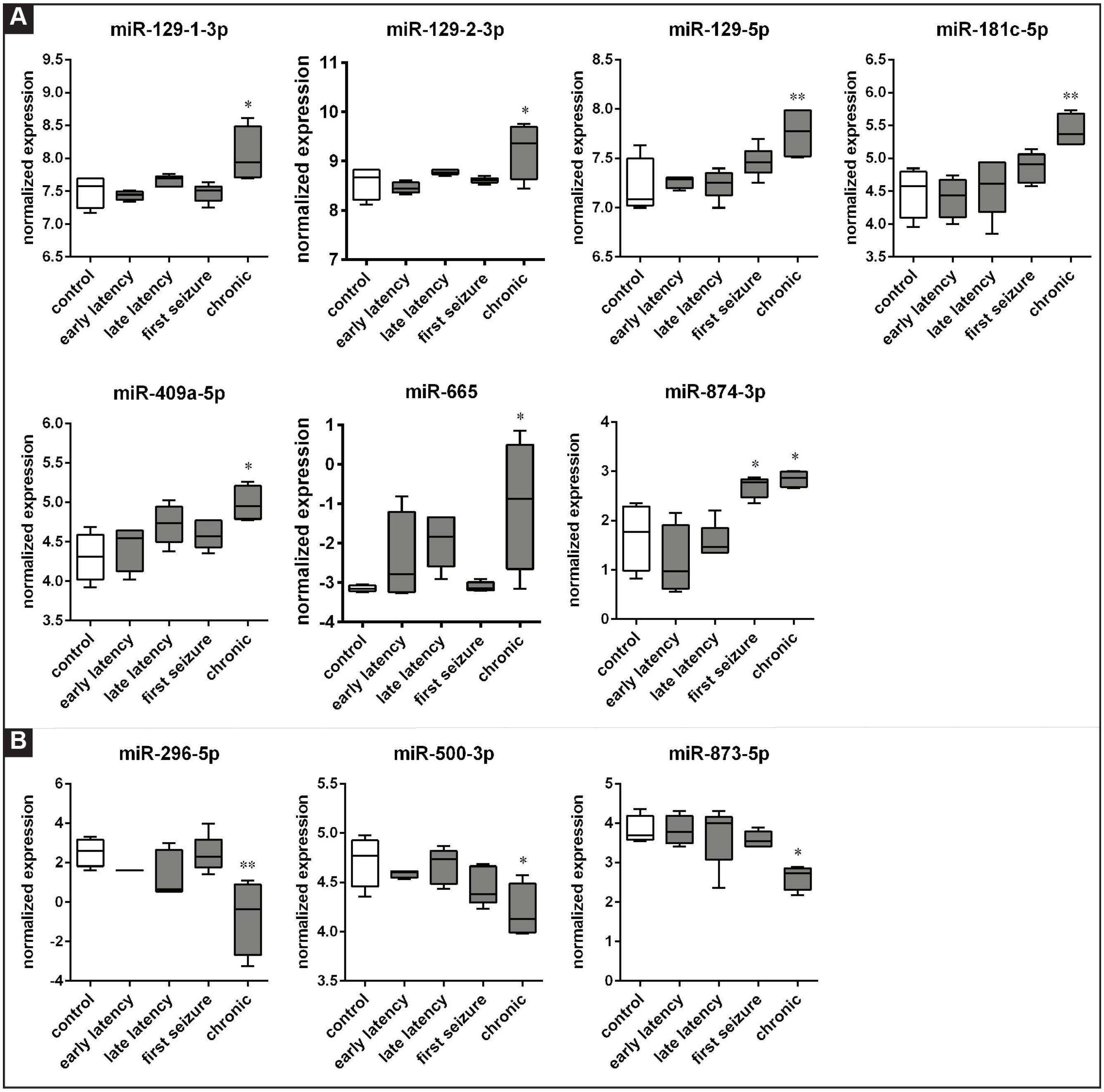

Supplementary Fig. S3. Deregulated miRNAs in the rat granule cell layer (GCL) during the chronic phase. The boxplots depict the time course of expression for 7 up-regulated (A) and 3 down-regulated (B) miRNAs identified as significant with a false discovery rate (FDR)<10% in the GCL by using one-way ANOVA. Each boxplot represents 4 animals. * p<0.05; ** p < 0.01; *** p<0.001; Tukey's test.

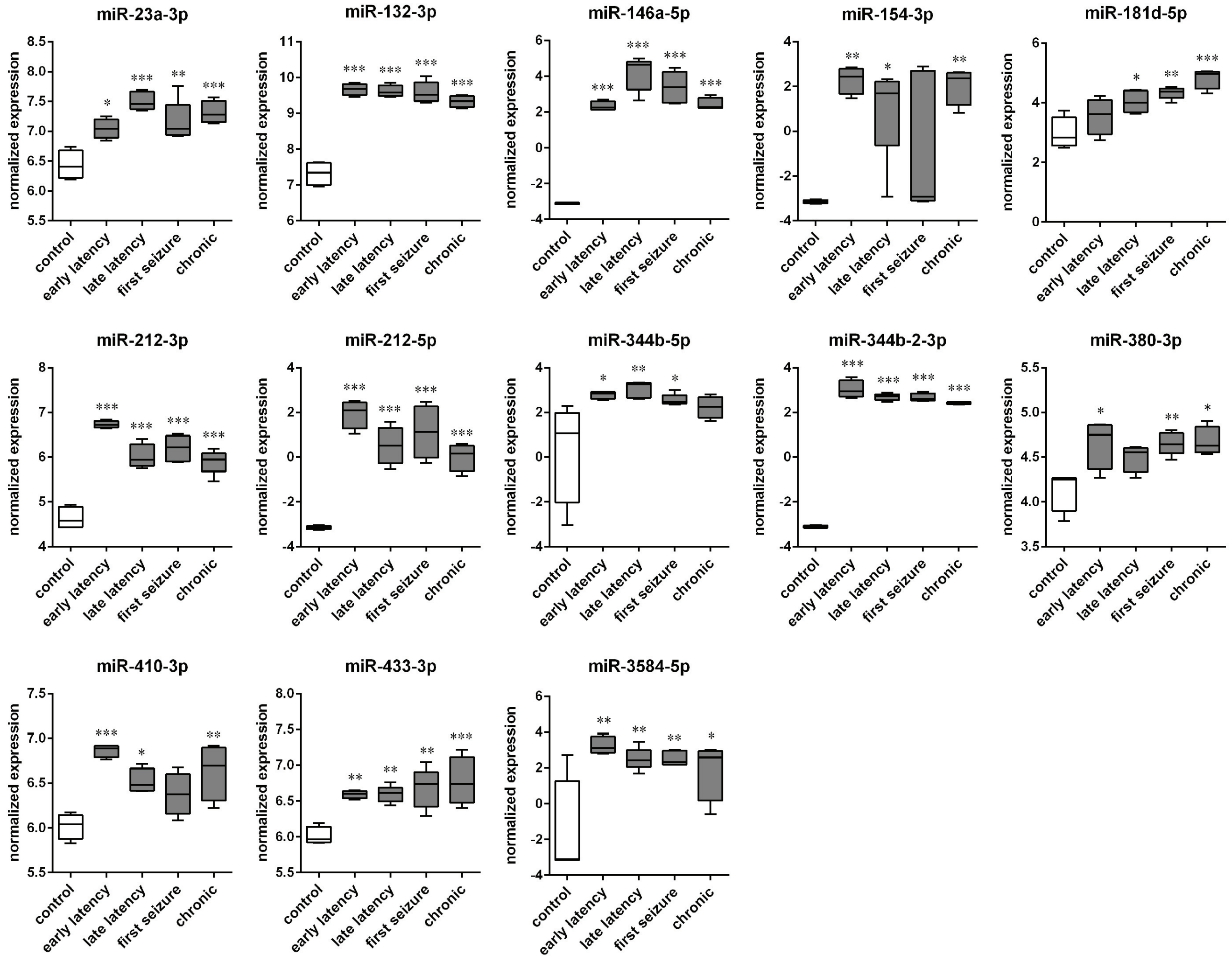

Supplementary Fig. S4. Up-regulated miRNAs in the rat granule cell layer (GCL) during latency. The boxplots depict the time course of expression for 13 of the miRNAs identified as significant with a false discovery rate (FDR)<10% in the GCL by using one-way ANOVA. Each boxplot represents 4 animals. * $p<0.05$; ** $p < 0.01$; *** $p<0.001$; Tukey's test.

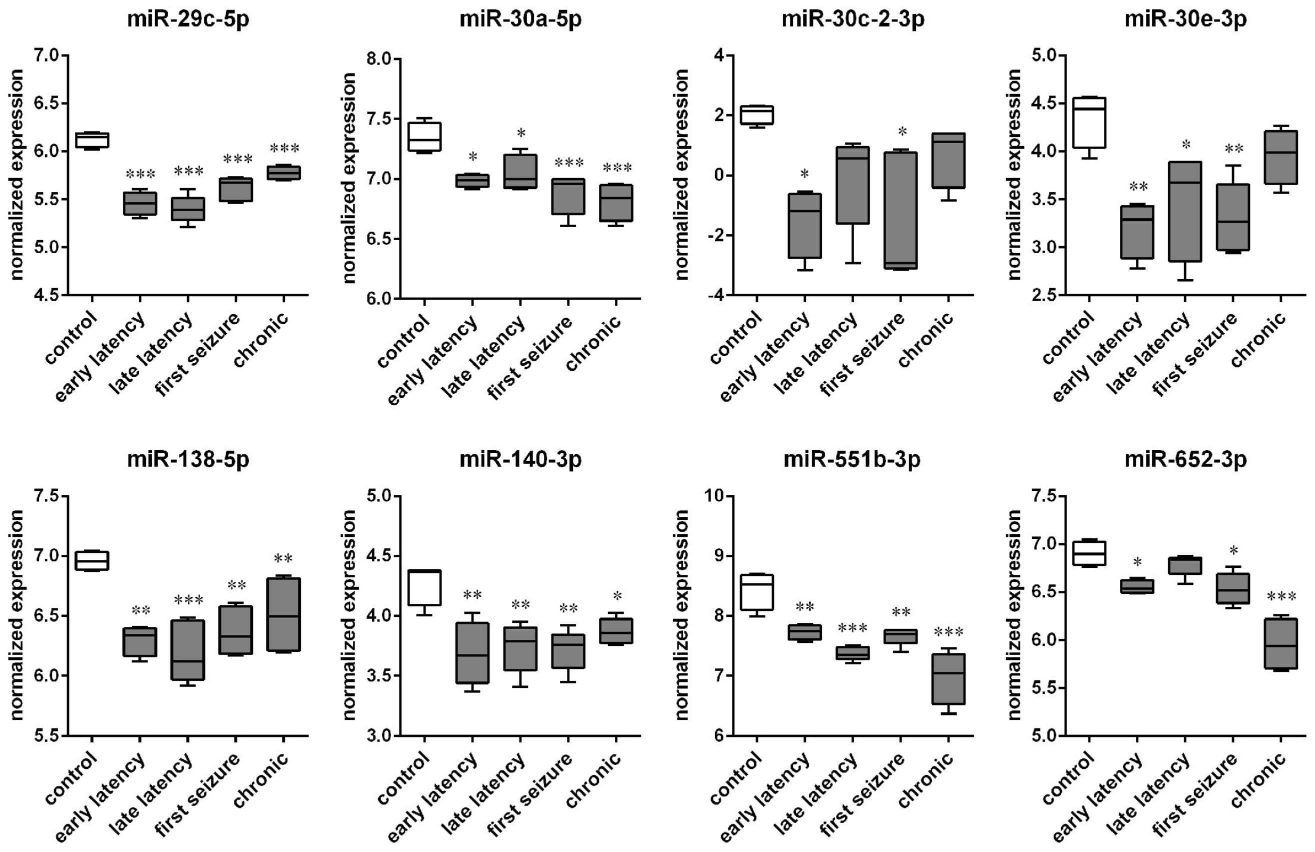

Supplementary Fig. S5. Down-regulated miRNAs in the rat granule cell layer (GCL) during latency. The boxplots depict the time course of expression for 8 of the miRNAs identified as significant with a false discovery rate (FDR)<10% in the GCL by using one-way ANOVA. Each boxplot represents 4 animals. * $p<0.05$; ** $p < 0.01$; *** $p<0.001$; Tukey's test.

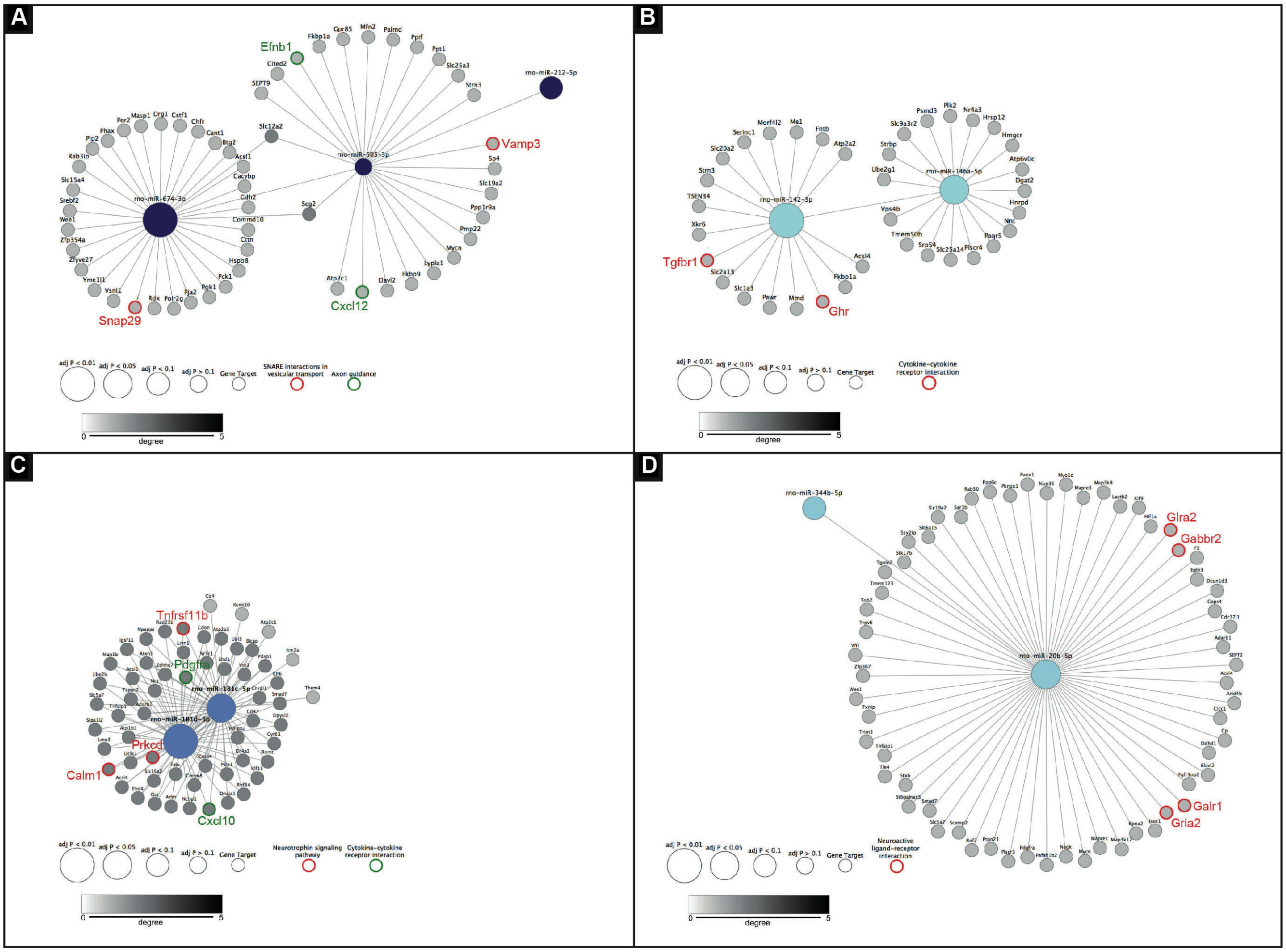

Supplementary Fig. S6. Network analysis. Predicted gene targets for each miRNA dys-regulated during latency (4 and 8 days after SE), obtained using the mirDB and TargetScan algorithms, were filtered with all miRNAs expressed in the dentate gyrus of rats (database GSE49850). (A) Cluster 1. (B) Cluster 3; (C) Cluster 4. (D) Cluster 5. Gene ontology (GO) and Kyoto Encyclopedia of Genes and Genomes (KEGG) analysis were performed using webgestalt, p<0.05.

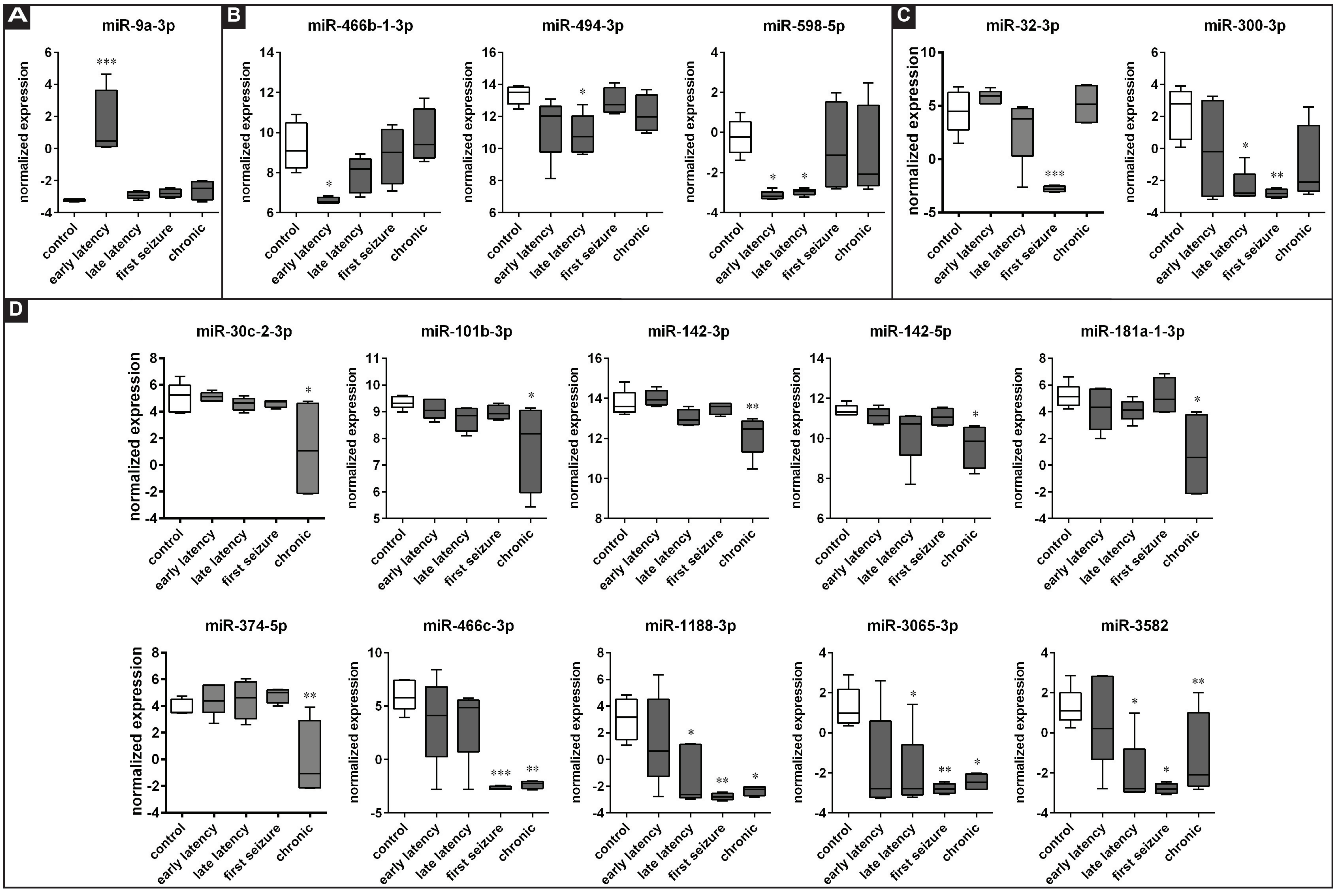

Supplementary Fig. S7. Deregulated miRNAs in the rat plasma during the all phases of the disease. The boxplots depict the time course for 16 of the miRNAs identified as significant by using one-way Anova. Each boxplot represents 5 animals. (A) miRNAs up-regulated during latency; (B) miRNAs down-regulated during latency; (C) miRNAs down-regulated in the first spontaneous seizure period; (D) miRNAs down regulated in the chronic period.
